# Effects of dietary supplements on androgenetic alopecia: a systematic review and network meta-analysis

**DOI:** 10.3389/fnut.2025.1719711

**Published:** 2026-01-05

**Authors:** Lei Zhou, Wenkang Zhu, Yan Chen

**Affiliations:** 1 Guang’anmen Hospital, China Academy of Chinese Medical Sciences, Beijing, China; 2 The First Clinical Medical College, Guangzhou Medical University, Guangzhou, China

**Keywords:** dietary supplements, androgenetic alopecia, hair density, efficacy, network meta-analysis

## Abstract

**Background:**

In recent years, androgenetic alopecia (AGA) has emerged as a significant public health concern due to its high prevalence and progressive nature. In addition to progressive scalp thinning and hair loss, patients often experience psychological distress and diminished quality of life. While standard treatments such as finasteride and minoxidil are effective, their side effects and adherence issues limit long-term use, making the exploration of safe and accessible intervention strategies essential. Dietary supplements, claimed to promote hair growth by inhibiting androgen pathways and improving the follicular microenvironment, have become an attractive adjunct for both clinicians and patients due to their low cost and ease of use. However, existing studies have limitations, including the diversity of supplements, small sample sizes, and the lack of direct comparisons among different supplements, making it unclear how they compare in terms of efficacy and safety. This study aims to use a network meta-analysis (NMA) to compare the effectiveness and safety of various dietary supplements based on outcomes such as hair density and terminal hair density, providing evidence-based support for clinical decision-making.

**Methods:**

A systematic search was conducted in English-language databases such as PubMed, Cochrane Library, Embase, and Web of Science for randomized controlled trials (RCTs) investigating the use of dietary supplements for treating AGA. Stata 16.0 software was used for network meta-analysis, and Revman 5.4 software was utilized for evaluating study quality and bias risk; additionally, the Grading of Recommendations Assessment, Development and Evaluation (GRADE) framework was applied to rate the certainty (quality) of evidence for the included studies.

**Results:**

A total of 19 RCTs involving 1,658 AGA patients were included, with 894 patients in the supplement group and 764 in the control group. Sixteen dietary supplements were investigated. Results showed that compared with placebo, standardized plant extracts (Nutrafol), apple extract (AMSbzs, AMS), tocotrienols, pumpkin seed oil (PSO), and a compound extract of Cistanche and Laminaria (MK-R7) significantly improved hair density. Multi-component supplements (ALRV5XR), standardized plant extracts (Nutrafol), and probiotics effectively increased terminal hair density. In blind doctor assessments, PSO, capsaicin-isoflavones (CI), saw palmetto extract (ESR), Omega 3&6, Lambdapil, Nutrafol, and Multi-component supplements (AGA-P) showed higher hair regeneration scores than placebo or conventional treatments. No significant differences were found between interventions in terms of the terminal-to-vellus hair ratio. Overall, all dietary supplements were found to be well-tolerated.

**Conclusion:**

Dietary supplements have a positive impact on hair density, terminal hair density, and blind doctor evaluations in patients with androgenetic alopecia, with good tolerability. They may serve as beneficial adjuncts or alternatives to conventional treatments. Future large-scale, high-quality RCTs are needed to verify these findings.

**Systematic review registration:**

https://www.crd.york.ac.uk/PROSPERO/view/CRD420251130173, Identifier CRD420251130173.

## 1 Introduction

Androgenetic alopecia (AGA) is the most common progressive hair disorder worldwide, characterized by gradual miniaturization of hair follicles and the transformation of terminal hairs into vellus hairs. It ultimately manifests as progressive scalp thinning or hair loss, severely impacting the patient’s appearance and quality of life (1). The epidemiological features of AGA exhibit significant age and gender differences: in men, the incidence increases progressively with age, with the prevalence of AGA nearing 50% in those over 50 years old (2). In women, AGA often presents as “female pattern hair loss” (diffuse thinning), with a significant increase in incidence after menopause due to hormonal fluctuations (3, 4). From a pathological perspective, the development of AGA results from a combination of genetic susceptibility and androgen metabolism disturbances. Individuals carrying AGA-susceptibility genes show a heightened sensitivity of their hair follicles to dihydrotestosterone (DHT). DHT, a potent metabolite of testosterone catalyzed by 5α-reductase, binds to androgen receptors in the hair follicles, initiating the process of follicular miniaturization. This manifests as a significant reduction in the anagen phase, prolonged telogen phase, and the gradual transformation of terminal hairs into vellus hairs. The diameter of the hair shaft becomes thinner, pigmentation decreases, and the result is visibly progressive hair loss (5, 6).

Currently, FDA-approved treatments for AGA include finasteride (which inhibits 5α-reductase to reduce DHT) and minoxidil (which dilates scalp blood vessels and activates hair follicles) (7, 8). Both treatments have definite efficacy and an overall acceptable safety profile. However, in some patients, influenced by inter-individual differences or tolerability, limited efficacy or decreased treatment adherence may still occur. Therefore, developing adjunctive interventions with complementary mechanisms of action and better tolerability is of great significance for improving the clinical treatment system for androgenetic alopecia.

Dietary supplements, due to their natural origins and multi-target mechanisms, have become a research hotspot for AGA interventions (9). Saw palmetto extract reduces DHT by inhibiting 5α-reductase (10); pumpkin seed oil, rich in β-sitosterol and plant sterol esters, exhibits similar anti-androgenic effects (11). Additionally, tocotrienols act as antioxidants to protect hair follicles (12), omega polyunsaturated fatty acids alleviate scalp inflammation (13), and probiotics have shown promising results in small-scale studies (14). However, existing studies are limited by small sample sizes and lack direct comparisons between different supplements, leaving their relative efficacy unclear.

Network meta-analysis (NMA) has distinct advantages over traditional meta-analysis as it allows for the comparison of multiple interventions within a single framework. Previous NMA studies have demonstrated that dietary supplements have clear positive effects on improving hair health in AGA patients, such as increasing hair density and improving external appearance (15). However, no NMA has been conducted on dietary supplements for treating AGA. This study represents the first comprehensive network meta-analysis aimed at systematically evaluating the efficacy and safety of dietary supplements for AGA, comparing their effectiveness in terms of hair density and follicular density.

A combined approach of systematic database search and manual supplementation was employed. Multiple English-language databases, including PubMed, Cochrane Library, Embase, and Web of Science, were searched to supplement the existing data. The search cutoff date was set to August 1, 2025.

The literature screening and data extraction were conducted independently by two researchers (LZ and WZ). Discrepancies were resolved through consultation with a third researcher (YC). The selected databases prioritized those commonly used in meta-analyses and covered all relevant RCTs. Additionally, we conducted comprehensive searches of published meta-analyses and review articles to ensure the completeness of the included studies.

The screening process was as follows: first, studies were screened by title and abstract, and irrelevant studies were excluded. The remaining studies were reviewed in full to determine if they met the inclusion criteria. The study selection strictly adhered to the Preferred Reporting Items for Systematic Reviews and Meta-Analyses (PRISMA) guidelines (16). The protocol for this meta-analysis has been registered on the Prospero platform (registration number: CRD420251130173).

## 2 Materials and methods

### 2.1 Inclusion and exclusion criteria

Inclusion criteria were based on the PICOS framework.

#### 2.1.1 Inclusion criteria

(1) The study design was a randomized controlled trial (RCT).
(2) The participants were aged ≥18 years.
(3) The population consisted of patients diagnosed with AGA by clinical presentation or dermoscopy. If a study included a small number of non-AGA hair loss patients (e.g., telogen effluvium, alopecia areata), it was included in the primary analysis only when AGA subgroup data were available separately; studies that did not explicitly specify the alopecia type were also included if the supplement’s mechanism clearly targeted AGA.
(4) The study reported at least one pre-specified outcome, including hair density, terminal hair density, blind doctor evaluations, and terminal-to-vellus hair ratio.
(5) The intervention group included at least one dietary supplement, and the control group was a placebo, conventional medication, or only lifestyle interventions.

In this study, “dietary supplements” referred to oral supplements designed to provide specific nutrients or bioactive compounds, including natural food/plant extracts (e.g., pumpkin seed oil, saw palmetto) and synthetic or composite nutritional formulations (e.g., Nutrafol, vitamin-mineral complexes). Only studies with RCTs supporting these interventions and evaluating outcomes such as hair density were included. Studies lacking relevant RCT data or with difficult-to-extract comparable outcome data were excluded. This definition and screening process aimed to reduce bias and ensure consistency in the evidence strength and comparability of the included interventions. The supplement categories are detailed in the Supplementary Table S1.

#### 2.1.2 Exclusion criteria

(1) Duplicate studies or publications.
(2) Animal studies.
(3) Studies involving pregnant women or children.
(4) Conference reports, letters, case reports, and studies with inaccessible full text.
(5) Non-randomized controlled trials.

### 2.2 Data extraction and quality assessment

Data extraction and quality assessment were conducted independently by two researchers. Any discrepancies in the evaluations were resolved through discussion, and if necessary, a third researcher was consulted to reach a consensus.

From each study, the following information was extracted: the first author, publication year, sample size, demographic characteristics of participants, intervention and control measures, treatment duration, inclusion criteria, and randomization methods. The risk of bias in the included RCTs was assessed using the Cochrane Risk of Bias tool (version 5.1.0) (17). Evaluate the risk of bias in RCTs included.

Assessment of evidence quality followed the GRADE approach (18–20), and was conducted independently by two reviewers, grading the certainty of evidence for each outcome as high, moderate, low, or very low. Randomized controlled trials were initially considered high-certainty evidence but could be downgraded based on five domains: risk of bias, inconsistency, indirectness, imprecision, and publication bias. Data analyses and evidence profiles were generated using GRADEpro GDT.

### 2.3 Statistical analysis

This study used Stata 16.0 and Stata 12.0 software with the mvmeta package for network meta-analysis and conventional meta-analysis. Quantitative data analysis was performed using “mean ± standard deviation” as the effect size indicator. Revman 5.4 software was used to evaluate the quality of the literature and assess bias risk. All effect measures were reported with 95% confidence intervals (CIs).

The *I*^2^ statistic was used to quantify heterogeneity. *I*^2^ values greater than 50% indicated significant heterogeneity. When *I*^2^ ≤ 50%, a fixed-effect model was used; otherwise, a random-effect model was applied. If significant heterogeneity was detected, a random-effect model was used to combine effect sizes, and subgroup analysis (e.g., by gender, intervention type) was conducted to explore the sources of heterogeneity. Additionally, sensitivity analysis was performed by excluding studies with high risk of bias or subgroups with high heterogeneity to assess the robustness of the results.

The cumulative ranking curve area (SUCRA) values (0–100%) were used to rank the effectiveness of the interventions. Higher SUCRA values indicate a higher probability of an intervention being the best treatment option. If studies provided standard deviations (SD) for baseline and follow-up absolute values but not for mean changes, missing values were imputed using the correlation method. For studies that did not report mean changes, the arithmetic difference between baseline and follow-up values was used. In multi-arm trials, data were processed and analyzed by combining subgroups or performing separate analyses for different interventions. For example, subgroups with the same type of intervention (e.g., dietary supplements with the same active ingredients) were merged for analysis to increase sample size and statistical power.

All continuous data were presented as “mean ± standard deviation,” and the data units were standardized. For quantitative outcomes such as hair density and terminal hair density, combined means and mean differences were calculated. The combined means were calculated using a weighted average, with weights determined by the sample sizes of the subgroups to ensure that larger subgroups had a greater influence on the overall results.

## 3 Results

### 3.1 Literature search results

**Figure 1:**
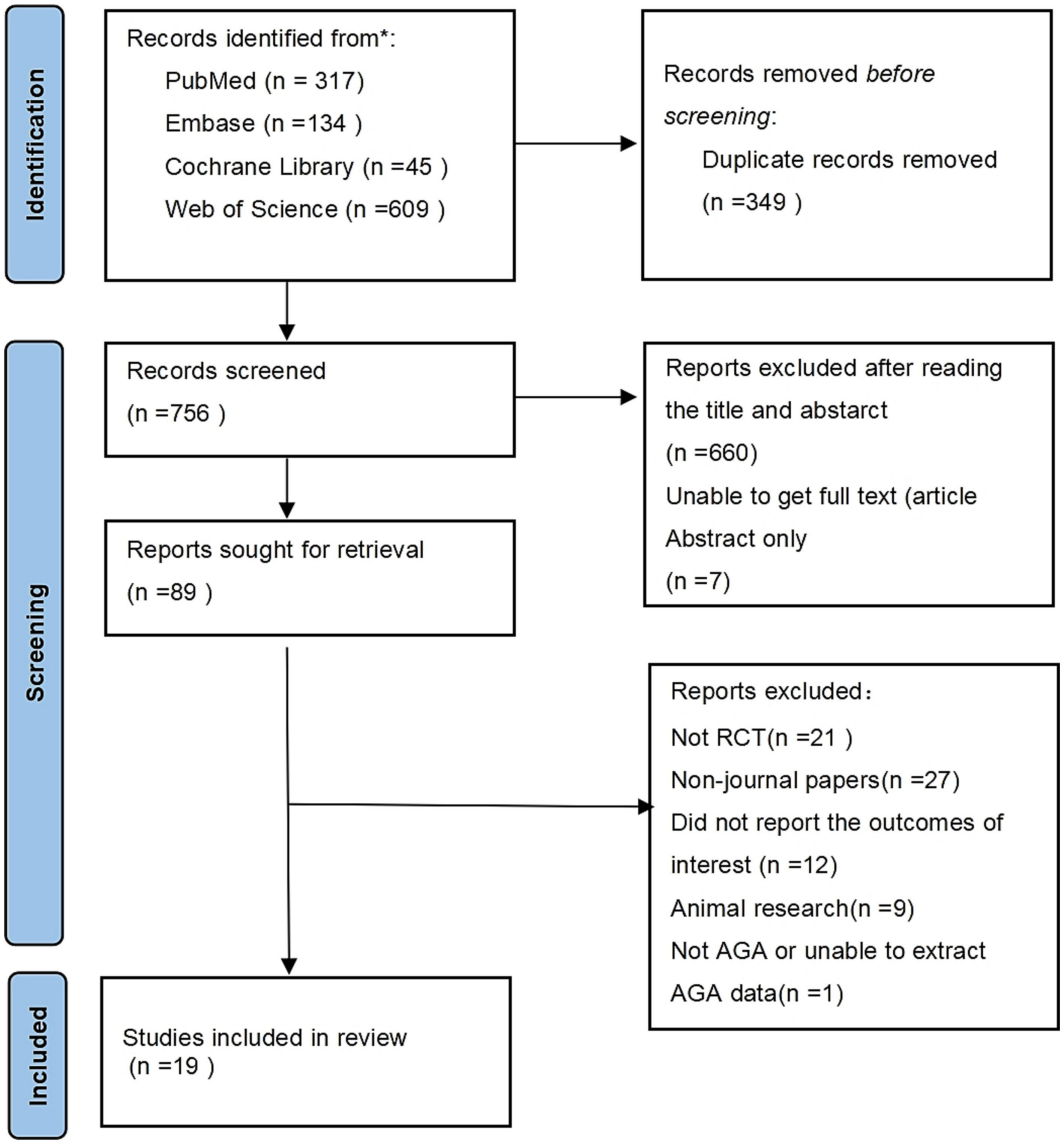
Document screening process and results.

A total of 1,105 potentially relevant studies were initially identified from the database search. After removing 349 duplicate studies, 756 studies were screened by title and abstract, with 667 studies excluded for not meeting the inclusion criteria. The remaining 89 studies underwent full-text evaluation. Finally, 19 RCTs were included in this meta-analysis (see Figure 1). The detailed search strategy and keywords are provided in the Supplementary Table S1.

### 3.2 Quality assessment of included studies

The quality of the 20 RCTs included in the meta-analysis was assessed independently by two researchers to ensure the reliability of the results. If discrepancies were found, consensus was reached through discussion, and a third researcher was consulted when necessary.

**Figure 2:**
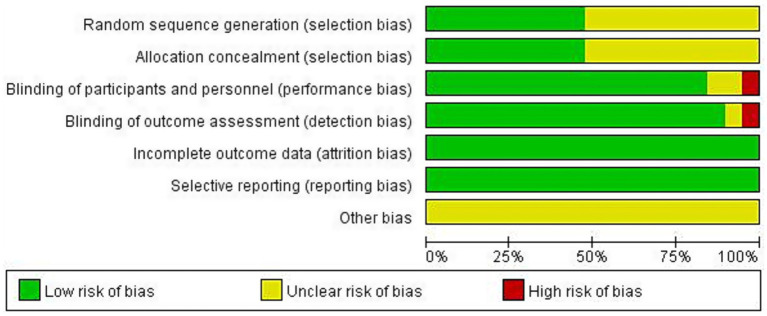
Risk of bias assessment included in the study.

In terms of random sequence generation, 9 studies used random number tables or computer-generated random sequences, and these were classified as low risk of bias (21–29); 10 studies only mentioned “random,” without providing clear details on the allocation method, and their bias risk was classified as “unclear” (12, 30–38). Regarding allocation concealment and blinding, 10 studies did not specify the allocation concealment methods, and bias risk was rated as “unclear” (12, 25, 27, 29–34, 37); 2 studies did not mention whether the participants and implementers were blinded, resulting in an “unclear” risk of bias (29, 31). One study did not mention blinding of outcome assessors, and was classified as “unclear” risk (32). One study used an open-label design, leading to a high bias risk classification (30). All studies provided complete data. After verifying the consistency between the methods and results sections of each study, the risk of selective reporting was assessed. It was found that all studies were classified as low-risk for selective reporting, with no significant omissions, although other potential sources of bias remained unclear. Bias risk assessments are shown in Figure 2, with detailed results available in the Supplementary Figure S1.

### 3.3 Basic information of included studies

This meta-analysis included 19 RCTs published between 2010 and 2025, involving 1,658 participants, with 894 in the supplement group and 764 in the control group. The studies covered 16 different dietary supplements, including plant/herbal formulations, marine extracts, amino acids, vitamins, minerals, and probiotics. The duration of the studies ranged from 16 to 32 weeks. Basic information about the included studies is provided in the Table 1 and Supplementary Table S2.

**Table 1:** Summary table in brief.

Supplement	Main components and typical dosages
ESR	① Betasitosterol (50 mg), saw palmetto extract (200 mg;), lecithin (50 mg), inositol (100 mg), phosphatidyl choline (25 mg), niacin (15 mg), biotin (100 μg); ② Dry plant extract (320 mg)
PSO	Capsules (400 mg); pumpkin seed oil (oleic acid, linoleic acid, linolenic acid)
FSE-M	Fenugreek seed extract (300 mg), niacinamide (18 mg), pantothenic acid (6 mg), multivitamins (145 mg), zinc (5 mg)
Tocotrienol	Mixed capsules (50 mg; 30.8% alphatocotrienol; 56.4% gammatocotrienol; 12.8% deltatocotrienol; 23 IUs alphatocopherol)
Omega 3&6	Fish oil (460 mg), blackcurrant seed oil (460 mg), vitamin E (5 mg), vitamin C (30 mg), lycopene (1 mg)
MK-R7	Extract of cistanche tubulosa (150 mg), extract of *Laminaria japonica* (50 mg)
AGA-P	*Serenoa repens* (320 mg), *Cucurbita pepo* (320 mg), L-Cystine (425 mg), vitamin C, zinc
Probiotics	Capsules (5 × 10^9^ CFU); *Lactobacillus*
CI	Capsaicin (6 mg); isoflavone (75 mg)
Viviscal	Vitamin C, zinc, AminoMar (shark powder and mollusk powder; 452.9 mg), horsetail extract (24.5 mg), flax seed extract (50 mg)
Nutrafol	Capsules of synergen complex, saw palmetto, maca, astaxanthin, curcumin, tocotrienols
AMSbzs	AMS with maltodextrins (400 mg), biotin (0.20 mg), selenomethionine (80.0 μg), zinc (21.0 mg)
AMS	Apple extract (from chlorogenic acid, procyanidin B2)
PPT5α	Saw palmetto (160 mg); pumpkin seed oil (100 mg); pomegranate extract (50 mg); zinc (10 mg); amino acids (50 mg L-cystine, 50 mg L-methionine), hydrolyzed collagen (250 mg); hyaluronic acid (25 mg), etc.
ALRV5XR	Capsules (842 mg); plant extracts (*Angelica sinensis*, *Rosmarinus officinalis*, etc.), multivitamins, minerals
Lambdapil	L-cystine (1,000 mg), S repens (100 mg), *Equisetum arvense* (7.14 mg), silicon (0.50 mg), zinc (10 mg), vitamin B3 (16 mg), vitamin B5 (6 mg), vitamin B6 (1.4 mg), D-biotin (50 μg), taurine (40 mg)

### 3.4 Network meta-analysis

#### 3.4.1 Hair density

**Figure 3:**
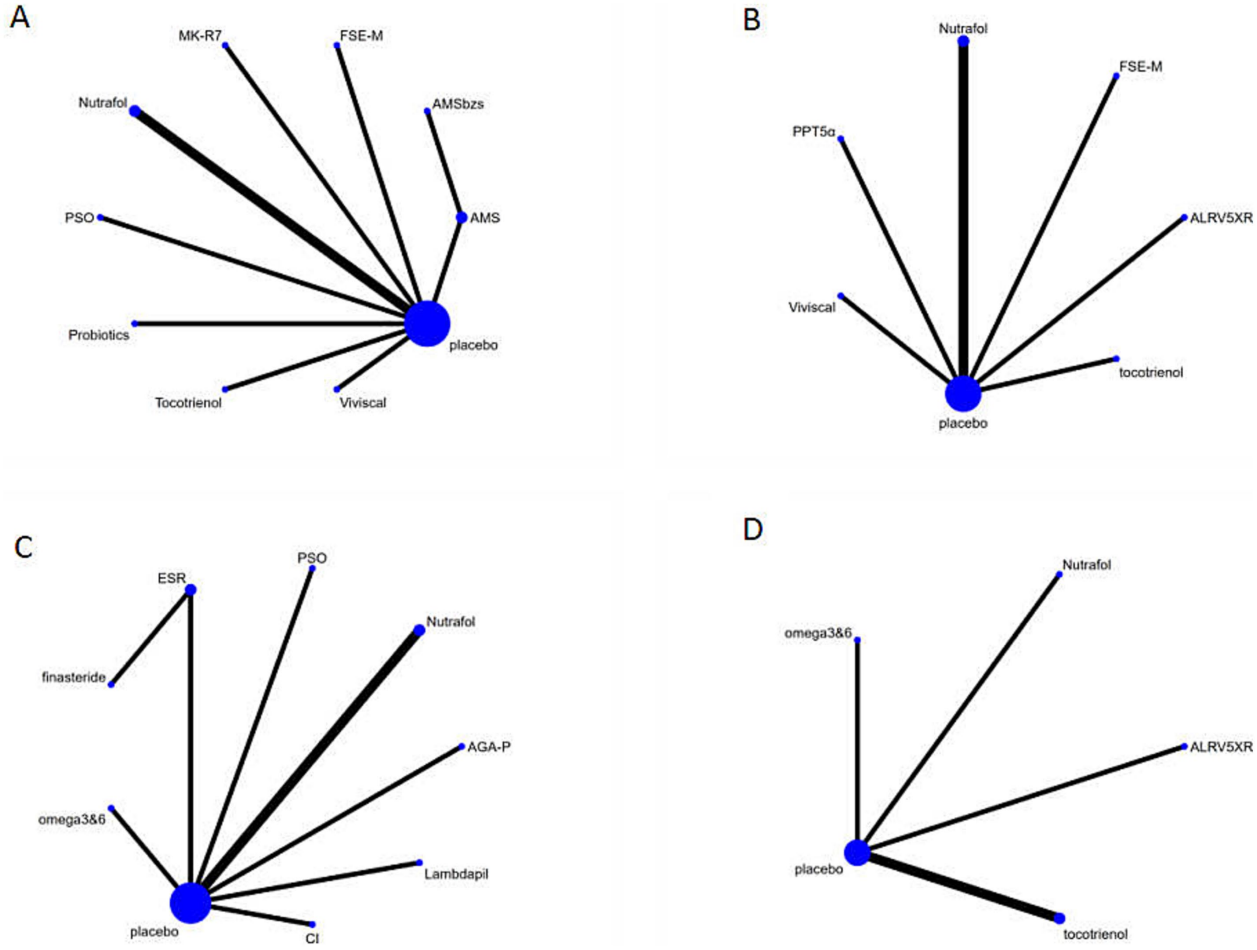
The evidence network of all papers on different treatments. **(A)** Hair density. **(B)** Terminal hair density. **(C)** Blind assessment by doctors. **(D)** Terminal hair/soft fur. The thickness of the lines represents the number of studies, and the sizes of the nodes indicate the total sample sizes for each treatment. In figures **A–C**, “Nutrafol” includes two studies.

The network of interventions included 10 pairwise comparisons, involving 9 different dietary supplements and 899 participants. The network relationships between these interventions are shown in Figure 3A. A consistency model was used throughout the network meta-analysis, and local inconsistency was evaluated using node splitting, while circular inconsistency was assessed to ensure overall network consistency. Heterogeneity testing was conducted to compare the differences between dietary supplements and the control group, and significant heterogeneity (*I*^2^ = 57.1%) was found, as shown in Supplementary Figure S2. Therefore, a random-effects model was applied.

The results showed that compared to placebo, the following dietary supplements significantly increased hair density: Nutrafol [SMD = 0.90 hairs/cm^2^, 95% CI (0.48, 1.33)], AMSbzs [SMD = 0.85 hairs/cm^2^, 95% CI (0.33, 1.38)], tocotrienols [SMD = 0.86 hairs/cm^2^, 95% CI (0.16, 1.56)], AMS [SMD = 0.81 hairs/cm^2^, 95% CI (0.36, 1.27)], pumpkin seed oil [PSO; SMD = 0.59 hairs/cm^2^, 95% CI (0.09, 1.09)], and MK-R7 [SMD = 0.58 hairs/cm^2^, 95% CI (0.17, 0.99)]. Group comparisons revealed that Nutrafol and AMSbzs performed better than probiotics [SMD = 0.69 hairs/cm^2^, 95% CI (0.13, 1.25)]. Detailed results of pairwise comparisons are available in Supplementary Table S3.

**Figure 4:**
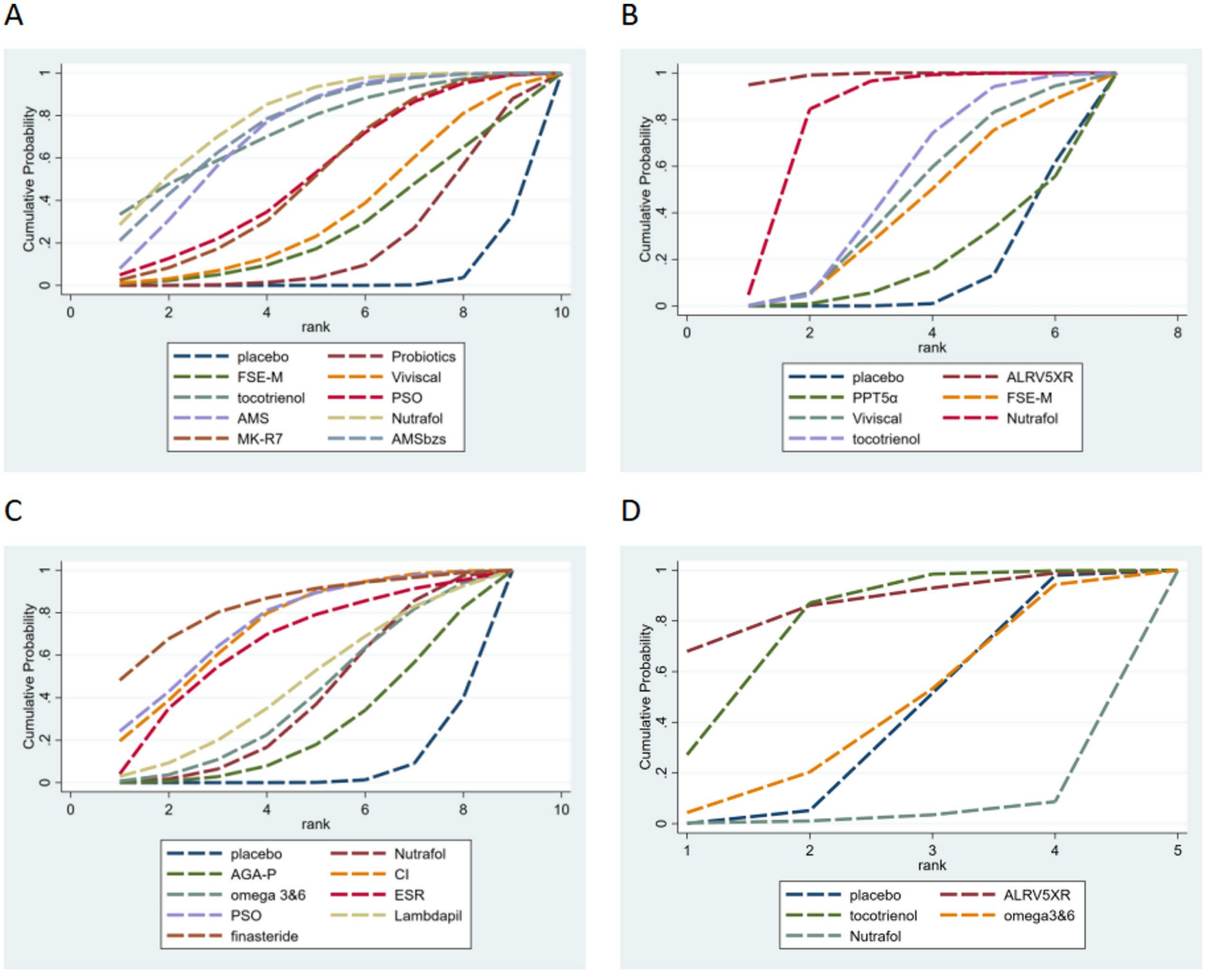
The SUCRA values for principal outcomes. **(A)** Hair density. **(B)** Terminal hair density. **(C)** Blind assessment by doctors. **(D)** Final hair/soft fur. In figures **A–C**, “Nutrafol” includes two studies.

In the ranking probability evaluation, Nutrafol had the largest area under the curve, indicating it may have the best treatment effect. AMSbzs and tocotrienols ranked just below Nutrafol in terms of treatment effect. The SUCRA values of other treatments are shown in Figure 4A and Table 2.

**Table 2:** The SUCRA values of each treatment modality.

Treatment	Hair density	Rank	Final hair density	Rank	Blind assessment by doctors	Rank	Final hair/soft fur	Rank
Lambdapil	—	—	—	—	46.0%	5	—	—
ALRV5XR	—	—	99.0%	1	—	—	86.70%	1
Probiotics	20.90%	9	51.80%	3	—	—	78.60%	2
FSE-M	30.10%	8	41.50%	5	—	—	—	—
AGA-P	—	—	—	—	25.60%	8	—	—
Tocotrienol	74.10%	3	—	—	—	—	—	—
PSO	52.60%	5	—	—	73.10%	2	—	—
CI	—	—	—	—	71.60%	3	—	—
Nutrafol	81.10%	1	80.80%	2	38.90%	7	3.10%	5
ESR	—	—	—	—	64.80%	4	—	—
PPT5α	—	—	18.30%	6	—	—	—	—
AMS	72.60%	4	—	—	—	—	—	—
Viviscal	35.70%	7	45.80%	4	—	—	—	—
MK-R7	52.30%	6	—	—	—	—	—	—
AMSbzs	76.60%	2	—	—	—	—	—	—
Omega 3&6	—	—	—	—	40.30%	6	42.60%	3
Finasteride	—	—	—	—	83.30%	1	—	—
Placebo	3.90%	10	12.70%	7	6.40%	9	39%	4

In the exploration of heterogeneity in the hair density outcome, the overall analysis showed significant heterogeneity (*I*^2^ = 57.1%). To identify the source of heterogeneity, subgroup analyses and meta-regression were conducted based on gender (male/female/mixed), intervention duration (16 weeks/24 weeks/32 weeks), and supplement type (single supplement/multi-component supplement). No significant effect-modifying factors were identified in any of these dimensions (subgroup differences and regression coefficients were not statistically significant), as shown in Supplementary Figures S3–S5 and Supplementary Table S7. Further sensitivity analysis by sequentially excluding studies with lower effect sizes revealed that excluding one study with a particularly low effect size (28), significantly reduced the overall heterogeneity to *I*^2^ = 17.1%, indicating that this study was the major source of heterogeneity. The detailed information and effect size data from this study are provided in Supplementary Figure S6.

Further extraction of core information and effect size data from this study: This study is a comparison between “apple extract ± biotin, selenium, zinc.” The intervention group (apple extract + biotin, selenium, zinc) showed an effect size relative to the control group (apple extract) of SMD = 0.04 hairs/cm^2^, 95% CI (−0.22, 0.30), which suggests a slight enhancement, but the effect size is significantly lower than that observed in other included studies.

#### 3.4.2 Terminal hair density

The network of interventions included 7 pairwise comparisons, involving 6 dietary supplements and 454 participants. The network relationships between these interventions are shown in Figure 3B. Consistency models were used throughout the network meta-analysis, and local inconsistency was evaluated using node splitting, while circular inconsistency was assessed to ensure overall network consistency. Heterogeneity testing revealed high heterogeneity (*I*^2^ = 60.5%) between the supplements and the control group, indicating substantial variability in effect sizes across studies, as shown in Supplementary Figure S7. Therefore, a random-effects model was applied.

Results showed that compared to placebo, the following dietary supplements significantly increased terminal hair density: ALRV5XR [SMD = 1.58 hairs/cm^2^, 95% CI (0.82, 2.34)], Nutrafol [SMD = 0.85 hairs/cm^2^, 95% CI (0.43, 1.27)], and Probiotics [SMD = 0.41 hairs/cm^2^, 95% CI (0.04, 0.78)]. No significant differences were observed in pairwise comparisons of the remaining interventions (*p* > 0.05). Group comparisons indicated that ALRV5XR performed better than most supplements (except Nutrafol), and Nutrafol performed better than PPT5α. Detailed results of pairwise comparisons are available in Supplementary Table S4.

In the ranking probability evaluation, ALRV5XR had the largest area under the curve, indicating it may have the best treatment effect. Nutrafol and Probiotics ranked just below ALRV5XR in terms of treatment effect. The SUCRA values of other treatments are shown in Figure 4B and Table 2.

Heterogeneity testing showed significant high heterogeneity (*I*^2^ = 60.5%) between dietary supplements and the control group, suggesting inconsistent effect sizes across studies. To identify the sources of heterogeneity, exploratory meta-regression was performed based on gender (male/female/mixed), intervention duration (16 weeks/24 weeks/32 weeks), and whether topical interventions were used in conjunction with oral supplements. The results showed no significant effect-modifying factors across these dimensions (all regression coefficients were not statistically significant), as shown in Supplementary Table S7.

Subsequently, these three variables were used as stratification factors, and direct pairwise comparisons of intervention effects within each stratum were performed using conventional meta-analysis methods. In the “non-combined topical intervention subgroup,” heterogeneity decreased to *I*^2^ = 25.2%, and the intervention showed statistically significant results [*p* < 0.001, SMD = 0.45, 95% CI (0.24–0.66)], suggesting that “whether topical active agents are combined” might be an important factor contributing to the overall heterogeneity. Detailed results are shown in Supplementary Figures S8–S10.

The sensitivity analysis further confirmed this finding: after excluding one studies that included “combined topical active agents” [SMD = −0.45, 95% CI (0.2, 0.7)], heterogeneity significantly decreased from *I*^2^ = 60.5% to *I*^2^ = 25.2%, consistent with the direction of the stratified analysis results, indicating that this factor has a stable influence on heterogeneity. Detailed results are shown in Supplementary Figure S11.

#### 3.4.3 Blind assessment by doctors

Hair growth and quality changes were assessed by blinded doctors using standardized methods. The network of interventions included 9 pairwise comparisons, involving 7 dietary supplements and 740 participants. The network relationships between these interventions are shown in Figure 3C. The heterogeneity test revealed high heterogeneity (*I*^2^ = 81.2%) between the supplements and the control group, as shown in Supplementary Figure S12. Therefore, a random-effects model was applied, and consistency models were used throughout the network meta-analysis, with node splitting and circular inconsistency tests for validation.

Results showed that, in blinded physician assessments of hair growth and quality, seven dietary supplements demonstrated significant advantages over conventional medication or placebo, namely pumpkin seed oil [PSO; RR = 1.72, 95% CI (0.15, 3.29)], capsaicin plus isoflavone [CI; RR = 1.73, 95% CI (0.31, 3.16)], ESR [RR = 1.68, 95% CI (−0.42, 3.79)], omega 3&6 [RR = 0.79, 95% CI (−0.24, 1.81)], Nutrafol [RR = 0.73, 95% CI (−0.02, 1.49)], Lambdapil [RR = 0.96, 95% CI (−0.39, 2.31)], and AGA-P [RR = 0.41, 95% CI (−0.56, 1.38)]; all differences were statistically significant (*p* < 0.05). Between-group comparisons indicated that all supplements were superior to GFM oral; PSO and CI were superior to Nutrafol, AGA-P, and omega 3&6. Detailed pairwise results are provided in Supplementary Table S5.

The results showed that in blind doctor evaluations, several dietary supplements, including PSO and CI, demonstrated significant advantages over placebo or conventional treatment (*p* < 0.05). SUCRA ranking showed that PSO had the largest area under the curve, suggesting that PSO is the best-performing supplement in terms of hair growth improvement, excluding the positive control drug finasteride. CI and ESR showed treatment effects just below PSO. The SUCRA values of other treatments are shown in Figure 4C and Table 2.

In this outcome, significant high heterogeneity (*I*^2^ = 80.2%) was observed. To identify the sources of heterogeneity, sensitivity analyses were performed by excluding high-bias-risk studies. Only one study was classified as high-risk for bias, and excluding this study did not significantly reduce heterogeneity, indicating that the high-bias-risk study was not the primary source of heterogeneity. Detailed results are shown in Supplementary Figure S13. After stratifying the subgroups by key variables such as “control scheme” and “gender,” The results showed that heterogeneity decreased markedly in the placebo-controlled subgroup, suggesting that the “control regimen” may be an important contributor to overall heterogeneity (see Supplementary Figures S14, S15). In meta-regression including covariates such as “assessment time,” “sex,” “differences in assessment scales,” and “control regimen,” the “control regimen” exhibited a significant association (*p* = 0.049), consistent with the direction of the stratified analyses and further supporting differences in control regimens as a source of heterogeneity as detailed in Supplementary Table S7.

In the 19 RCTs evaluated, 53% (10 studies) were judged to have an unclear risk of bias in random sequence generation. Only 47% (9 studies) clearly described the allocation concealment methods, while 15% (3 studies) did not report blinding details. The missing information may introduce potential selection bias and performance bias, contributing to the overall heterogeneity.

#### 3.4.4 Terminal hair-to-vellus hair ratio

Five studies explored the effect of dietary supplements on the terminal-to-vellus hair ratio, involving four intervention schemes and 221 participants. The network relationships between these interventions are shown in Figure 3D. The heterogeneity test revealed low heterogeneity (*I*^2^ = 46.1%) between the dietary supplements and the control group, as shown in Supplementary Figure S16. Therefore, a fixed-effect model was applied, and consistency models were used throughout the network meta-analysis.

The results showed that in regulating the terminal-to-vellus hair ratio, six dietary supplements exhibited certain trends when compared to conventional treatment or placebo, but the differences were not statistically significant (*p* > 0.05). Group comparisons showed that ALRV5XR and Probiotics performed better than Nutrafol. Detailed results of pairwise comparisons are available in Supplementary Table S6.

In the SUCRA ranking probability evaluation, ALRV5XR had the largest area under the curve, suggesting that it was the best-performing supplement in terms of the terminal-to-vellus hair ratio. Probiotics and Omega 3&6 followed closely behind ALRV5XR. The SUCRA values of other treatments are shown in Figure 4D and Table 2.

### 3.5 Subgroup analysis

#### 3.5.1 Control group subgroup analysis

First, conventional meta-analysis methods were used to perform subgroup analyses of the different control groups. The interventions included two distinct control groups: (1) placebo or no intervention, and (2) conventional treatment or finasteride. The subgroup analysis results showed that, compared to placebo or no intervention, dietary supplements significantly improved blind doctor evaluations, with statistically significant differences (RR = 2.21, *p* = 0.005) (see Supplementary Figure S17). However, compared to finasteride or conventional treatment, dietary supplements only showed a trend of increased blind doctor evaluation, with no statistically significant difference (RR = 0.56, *p* = 0.587) (see Supplementary Figure S18).

The possible reason for this difference is that compared to placebo or no intervention, the absolute effect of supplements is easier to observe. In contrast, in the presence of an active control (which has a strong effect and works quickly), supplements mostly show incremental effects, which are harder to achieve statistically significant differences. This indicates that while dietary supplements do offer visible clinical benefits over placebo, their overall effect is not as potent as first-line prescription drugs. Clinically, supplements should be used as adjuncts to standard treatments, preferably combined with conventional therapies to maximize observable effects.

#### 3.5.2 Subgroup analysis by female age

Furthermore, subgroup analyses were performed based on the age characteristics of the population included in the dietary supplement studies, with 3 studies focusing on women and subdividing them into menopause and non-menopause subgroups. The results showed that in the menopause group, the terminal hair density level after dietary supplement intervention was significantly higher than that of placebo (SMD = 0.97, *p* < 0.001), see Supplementary Figure S19. However, in the non-menopause group, the dietary supplement showed only a trend of increased terminal hair density compared to placebo, with no statistically significant difference (SMD = 0.33, *p* = 0.334), see Supplementary Figure S20.

The difference may be due to the decline in estrogen levels in postmenopausal women, leading to a relative increase in androgen dominance, accompanied by increased oxidative stress and low-grade inflammation, as well as micronutrient deficiencies. As a result, interventions with anti-androgenic, antioxidant, and nutritional correction mechanisms have a greater “reversible space,” making the improvement in terminal hair density more significant. Based on this, future studies and clinical practice should prioritize postmenopausal women as the beneficiary group for nutritional supplements, and further targeted research should be conducted to validate and optimize intervention protocols for this population.

#### 3.5.3 Gender subgroup analysis

Moreover, considering that the efficacy of supplements may be influenced by gender-related physiological characteristics (such as hormone levels and follicular sensitivity to nutrients), this study conducted a subgroup analysis based on “gender” to determine whether there are gender-specific differences in the efficacy of supplements in improving terminal hair density. A conventional meta-analysis was performed on gender. A total of 5 studies were included in the gender subgroup analysis. The results of the subgroup analysis showed that in the female subgroup (3 studies), the effect of supplements on improving terminal hair density was statistically significant (SMD = 0.56, *p* = 0.008) (see Supplementary Figure S21). In the male subgroup (2 studies), the effect size was higher than that in the female subgroup but did not achieve statistical significance (SMD = 0.95, *p* = 0.136) (see Supplementary Figure S22).

The observed difference suggests that female AGA patients show more stable signals of benefit in terminal hair density outcomes. The pathogenesis of female AGA is more complex than in males, necessitating personalized, multidisciplinary, and standardized comprehensive treatments. The intervention methods should also have multiple effects. It is important to note that although the male subgroup had a higher effect size, only two studies were included in this subgroup, and the small sample size led to insufficient statistical power, making it difficult to exclude the possibility of “false negative” results. These factors may have caused fluctuations in the effect size across studies. Therefore, these differences should be interpreted with caution. Future research should include more high-quality studies with male participants and further control for potential confounders within same-gender subgroups to improve the precision and reliability of the results.

### 3.6 Sensitivity analysis

We conducted sensitivity analyses for all primary outcomes restricted to studies at low risk of bias. After excluding 13 trials rated as having moderate-to-high risk of bias, and recognizing the potential for residual clinical heterogeneity, we re-estimated pooled effects using random-effects models. The results showed no substantive changes in the direction or magnitude of effects for the primary outcomes—hair density, terminal hair density, blinded physician assessment, and the terminal/vellus hair ratio. Differences remained statistically significant for hair density (*p* = 0.017) and terminal hair density (*p* = 0.012). These findings indicate that the main conclusions of this study were not materially influenced by lower-quality trials and that the overall results are robust. See Supplementary Figures S23–S26.

### 3.7 Adverse events

Adverse events (AE) were systematically extracted from the RCTs included in this study for safety analysis. The results showed that among the 19 studies included in the analysis, 11 interventions reported adverse events, all of which were mild symptoms such as bloating, diarrhea, and itching. These symptoms were typically relieved without the need for medical intervention. The results are shown in Tables 3, 4.

**Table 3:** Adverse events of different treatments.

Treatment	Diarrhea (*n*)	Flatulence (*n*)	Itchy scalp (*n*)	Generalized pruritus (*n*)	Constipation (*n*)	Loss of appetite (*n*)	Gastrointestinal discomfort (*n*)	Stomachache (*n*)
Lambdapil	—	1	—	—	—	—	—	—
ALRV5XR	—	—	1	—	—	—	—	—
Probiotics	—	2	—	—	—	—	—	—
FSE-M	—	2	—	—	1	—	—	—
PSO	—	—	—	1	—	—	1	—
Nutrafol	—	1	—	—	—	—	3	—
ESR	—	—	—	—	—	1	—	—
PPT5α	1	—	—	—	—	—	—	—
AMSbzs	—	—	—	—	—	—	1	2
AMS	—	—	—	—	—	—	3	2
MK-R7	—	1	—	—	—	—	—	—

**Table 4:** The incidence rate, duration and termination of adverse events related to supplements.

Intervention	Cases (*n*, %)	Duration (days)	Treatment discontinuation (yes/no)
Lambdapil	1 (2.8%)	NI	Yes
Probiotics	2 (1.4%)	7–14	No
FSE-M	3 (5.3%)	3	No
PSO	2 (2.6%)	NI	No
Nutrafol	4 (1.8%)	NI	No
ESR	1 (0.8%)	NI	No
PPT5α	1 (2.1%)	NI	Yes
AMSbzs	3 (1.2%)	NI	No
AMS	5 (6.2%)	NI	No
MK-R7	1 (1.0%)	NI	No

Although the overall incidence of adverse events was low and the symptoms were generally mild, the completeness of safety data reporting should still be emphasized. In this study, the Nutrafol group reported 1 case of bloating and 3 cases of stomach discomfort, possibly related to individual sensitivity to the natural active substances in plant ingredients (e.g., fatty acids in saw palmetto, steroid saponins in South African wild ginger), which could irritate the gastrointestinal mucosa. The AMS group reported 3 cases of stomach discomfort and 2 cases of stomach pain, with symptoms not reaching the level of clinical intervention, and were initially considered to be mild irritation related to the ingredients. The PSO group reported 1 case of generalized itching and 1 case of abdominal discomfort, which may be related to the bioavailability and individual tolerance of lipid-soluble ingredients.

Additionally, there are significant limitations in the safety data: most studies only focused on short-term safety (adverse events during the study period), leading to a lack of comprehensive safety data. While the existing evidence suggests that dietary supplements are generally safe, with only mild gastrointestinal adverse events in some patients, the interpretation of this conclusion should be cautious, especially for vulnerable populations such as the elderly, children, and pregnant women. There is insufficient data to support the safety of these supplements for these groups. Future studies should provide detailed, standardized, and comprehensive reports on the potential risks of dietary supplements to provide more reliable evidence for their safe clinical application.

### 3.8 Publication bias and assessment of evidence quality

**Figure 5:**
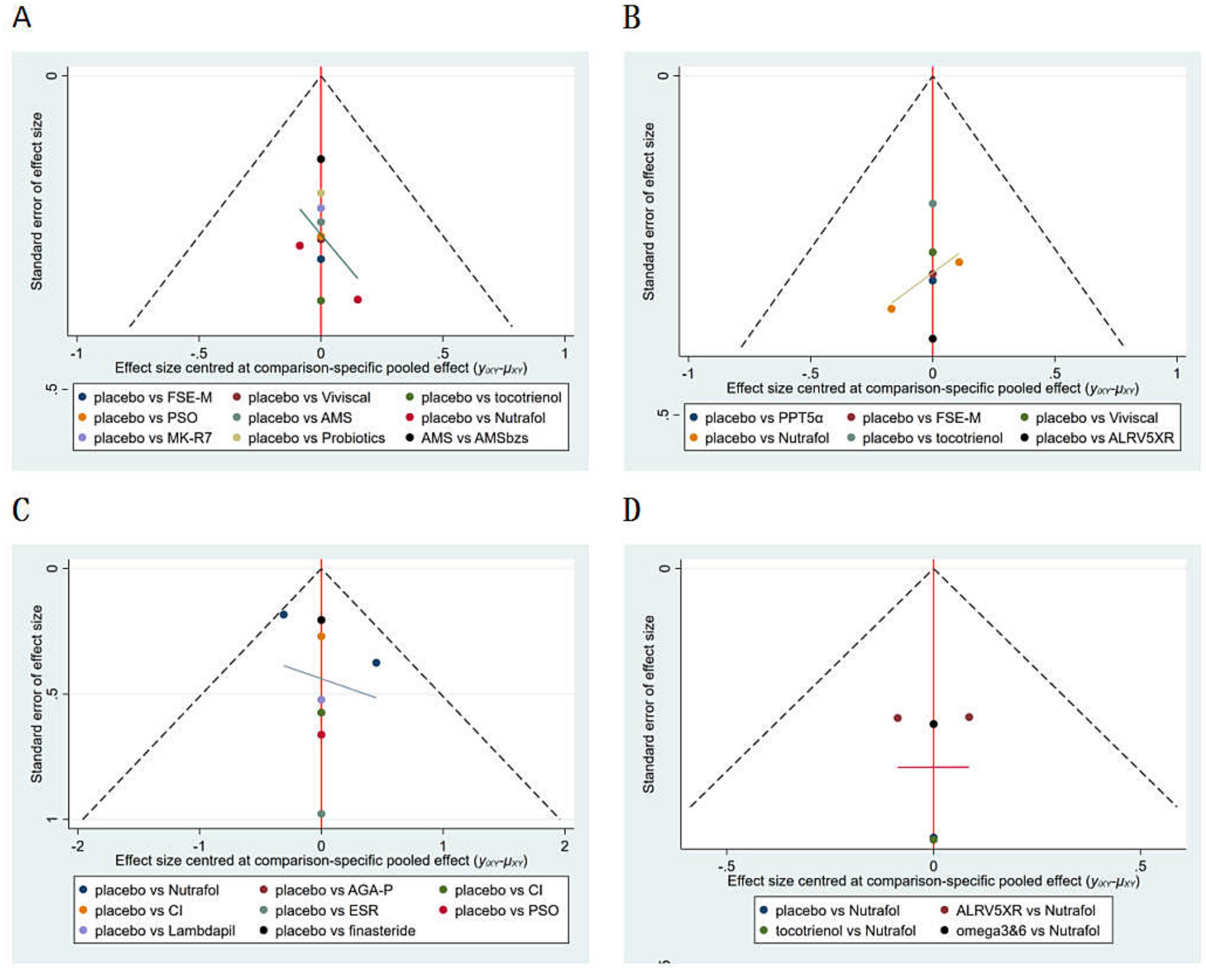
Funnel plot for publication bias in all outcomes. **(A)** Hair density. **(B)** Terminal hair density. **(C)** Blind assessment by doctors. **(D)** Final hair/soft fur. In figures **A–C**, “Nutrafol” includes two studies.

Funnel plots comparing terminal hair density and terminal-to-vellus hair ratio indicated that study data points were symmetrically distributed around the midline, suggesting low publication bias for these outcomes. In contrast, the funnel plots for hair density、terminal hair Density and blind doctor evaluations were asymmetric, suggesting potential publication bias or small sample effects. As shown in Figure 5, in the hair density and terminal hair Density outcome, most studies had a sample size of less than 100, which results in low statistical power and may lead to small effects being misjudged as “no difference,” causing negative results to remain unpublished. Similarly, for the blind doctor evaluation outcome, this is often considered a “secondary outcome,” with only positive results being more likely to be accepted by journals, while negative results are often omitted. Additionally, many studies were single-center, small-sample studies (*n* < 100), with insufficient statistical power, which limits the stability of the results and contributes to the bias. Therefore, the interpretation of the results from this meta-analysis should be cautious, especially for conclusions drawn from a small number of studies. It is more prudent to validate and consolidate these conclusions after more large-scale, high-quality studies are included.

Using the GRADE approach to assess certainty of evidence, the results indicated that the outcomes “hair density” and “terminal/soft hair ratio” were rated as moderate certainty, whereas “terminal hair density” and “blinded physician assessment” were rated as low certainty. The principal factors influencing these ratings included: relatively limited sample sizes in studies of dietary supplements and few observed events, resulting in wide confidence intervals; substantial heterogeneity for certain outcomes, which undermined reliability; and the predominance of “intervention vs. placebo” comparisons without head-to-head trials, necessitating reliance on indirect comparisons and further constraining the overall certainty of evidence. The results are shown in Table 5.

**Table 5:** The SUCRA values of each treatment modality.

Certainty assessment							Effect	Quality
No. of studies	Design	Risk of bias	Inconsistency	Indirectness	Imprecision	Other considerations	Effect size (95% CI)	
Hair density								
10	RCTS	No serious	No serious	No serious	No serious	Serious	SMD = 0.52, 95% CI: 0.29, 1.32	Moderate
Final hair density								
7	RCTS	Serious	No serious	No serious	No serious	Serious	SMD = 0.58, 95% CI: 0.24, 0.91	Low
Blind assessment by doctors								
9	RCTS	No serious	Serious	No serious	No serious	Serious	RR = 2, 95% CI: 1.23, 3.27	Low
Final hair/soft fur								
5	RCTS	No serious	No serious	No serious	Serious	No serious	SMD = 0.14, 95% CI: −0.05, 0.34	Moderate

RCTs, randomized controlled trials; CI, confidence interval; MD, standardized mean difference; RR, relative risk. GRADE working group grades of evidence: high quality, further research is very unlikely to change our confidence in the estimate of effect; moderate quality, further research is likely to have an important impact on our confidence in the estimate of effect and may change the estimate; low quality, further research is very likely to have an important impact on our confidence in the estimate of effect and is likely to change the estimate; very low quality, we are very uncertain about the estimate.

## 4 Discussion

### 4.1 Main results summary

This study is the first network meta-analysis on dietary supplements for treating androgenetic alopecia, with its core value being the ability to compare the relative efficacy of different supplement treatments. The study covered 16 interventions, 19 RCTs, and 1,658 participants, quantifying objective outcomes such as hair density and terminal hair density, along with the subjective outcomes of blind doctor evaluations, generating a SUCRA efficacy ranking to guide clinical decisions. The network meta-analysis showed.

For hair density improvement, six interventions, including Nutrafol, AMSbzs, AMS, tocotrienol, PSO, and MK-R7, significantly outperformed placebo (*p* < 0.05). The SUCRA ranking of these treatments indicated Nutrafol as the most effective (80.8%), suggesting it has the best potential for application in improving hair density.

For terminal hair density improvement, ALRV5XR, Nutrafol, and Probiotics were significantly better than placebo (*p* < 0.05), with ALRV5XR (99.0%) ranking first. Further subgroup analysis found that combining dietary supplements with topical active agents led to a more significant improvement in terminal hair density.

In the blind doctor evaluations, various dietary supplements, including PSO and CI, demonstrated significant advantages over the control group (*p* < 0.05). SUCRA ranking showed that PSO performed the best (73.1%), suggesting superior improvement in hair growth in the doctors’ assessments, aside from the positive control drug finasteride.

For the terminal-to-vellus hair ratio, no significant statistical differences were observed among dietary supplements and the control group, but the SUCRA ranking showed ALRV5XR, Probiotics, and Omega 3&6 leading, suggesting a slight improvement trend, requiring further verification with larger sample sizes.

In terms of safety, the dietary supplements included in this study generally had good tolerability, with 11 interventions reporting mild adverse events such as bloating, diarrhea, and slight itching, with no severe adverse events. However, many studies were short-term (≤24 weeks), lacking systematic safety data for long-term use.

Our subgroup analyses and meta-regression further examined how intervention modality and population characteristics contributed to treatment heterogeneity. Overall, most pre-specified dimensions (e.g., participant sex, treatment duration, and whether the supplement was single- or multi-component) did not show significant differences in effect size in the aggregate analyses, suggesting no marked inconsistency in supplement efficacy across subgroups given the current evidence. Nevertheless, a few factors may exert moderating effects: when dietary supplements were combined with topical therapy, improvements in terminal hair density were more pronounced, indicating potential synergy between systemic and local treatments; with respect to comparator type, supplements significantly improved blinded physician-rated outcomes versus placebo or no treatment, but showed no clear advantage over active conventional therapies (e.g., finasteride), implying that supplements are better suited as adjuncts rather than replacements for standard care; sex-stratified analyses showed a statistically significant benefit of supplements on terminal hair density in women, whereas the effect size in men—although numerically larger—did not reach significance, likely owing to smaller male sample sizes, indicating a clearer signal of benefit in women (particularly peri-/postmenopausal women) and comparatively limited evidence in men. Studies that stratified women by menopausal status further suggested that terminal hair density improved significantly versus placebo among menopausal women, while non-menopausal women exhibited only a non-significant upward trend. Taken together, these findings imply that dietary supplements may be more effective in specific populations or scenarios (e.g., female patients—especially those who are menopausal—and when used in combination with topical therapy). However, because some subgroups included few studies, non-significant findings may reflect limited statistical power; thus, the subgroup results should be interpreted with caution.

### 4.2 Comparison with other studies

Previous meta-analyses and systematic reviews on AGA have mostly focused on the effectiveness of conventional drug interventions or summarized the evidence on the use of dietary supplements. For example, a network meta-analysis in 2025 explored the effects of nine non-prescription treatments for male androgenetic alopecia (7). A 2023 systematic review examined the application of various dietary supplements in non-scarring hair loss (39).

The results of this study are consistent with the conclusions from the “Expert Consensus on the Clinical Application of Oral Nutritional Supplements in Hair Loss Treatment,” which confirms that oral nutritional supplements can effectively improve hair loss symptoms. Furthermore, the consensus recommends multi-component nutritional supplements over single-ingredient supplements for broader applicability, and also recommends combining them with other treatment modalities (40). The findings of this study align closely with this view, providing further evidence-based support for the clinical use of oral nutritional supplements.

### 4.3 Interpretation of results

As a common clinical progressive hair disorder, the onset of AGA is closely related to follicular miniaturization mediated by dihydrotestosterone (DHT) (41, 42). Although synthetic drugs like finasteride are used to treat AGA, the reported adverse events and the growing demand for natural interventions have driven research into alternatives such as plant extracts and multi-component nutritional supplements (43, 44). Investigating the mechanisms and clinical efficacy of such natural interventions is crucial for enriching treatment strategies for AGA and enhancing patient adherence (15).

Lowering DHT is a key strategy in AGA management. Studies have shown that maintaining DHT levels within a normal range can prevent follicular miniaturization and promote terminal hair growth to reduce hair loss. If DHT levels remain uncontrolled, hair loss can progress rapidly (41, 45). Common plant extract dietary supplements, such as saw palmetto and South African wild ginger, can reduce DHT production by inhibiting 5α-reductase (46). This network meta-analysis confirmed that multi-component plant extract supplements, such as Nutrafol, which contains saw palmetto, curcumin, and tocotrienols, are effective for AGA treatment. Among these, curcumin exerts anti-inflammatory and antioxidant effects, downregulating androgen receptors in hair follicles; South African wild ginger reduces cortisol to alleviate stress-related hair loss; and saw palmetto inhibits 5α-reductase (46). A study involving 100 male AGA patients showed that taking 320 mg of saw palmetto daily for two years improved hair loss in 38% of patients (though lower than finasteride’s 68% effectiveness, but with fewer side effects) and blocked the binding of DHT to hair follicle receptors (38). Tocotrienols protect hair follicles, and a randomized controlled trial demonstrated that taking tocotrienols for 8 months increased hair count by about 34.5% compared to baseline (12). Nutrafol Women’s Balance, an improved formulation for peri-menopausal women, also contains maca (which regulates hormones and alleviates menopausal symptoms) and astaxanthin (which enhances follicular antioxidant and anti-inflammatory effects), in addition to higher saw palmetto content. Clinical studies show that after 6 months of intervention, 73% of menopausal women experienced improved hair growth. Therefore, Nutrafol and its improved formulation, with their multi-mechanistic synergy, perform well across different populations and are ranked highly for improving hair density in this network meta-analysis.

ALRV5XR works through a synergistic combination of plant extracts, vitamins, and minerals, intervening in multiple mechanisms to treat androgenetic alopecia. Its plant ingredients, such as *Angelica sinensis*, *Laminaria*, and *Eclipta prostrata*, improve follicular microcirculation, eliminate oxidative damage, activate hair follicle stem cells, and inhibit 5α-reductase to reduce DHT production, thus extending the hair growth cycle (47–49). Vitamins B6, biotin, and vitamin D regulate androgen balance in the body, promote keratin synthesis (the core component of hair), and activate hair follicle growth factors, providing fundamental support for hair growth (50, 51). Zinc and selenium protect hair follicles from damage through antioxidant and anti-inflammatory effects, promoting follicular regeneration. Meanwhile, the supporting topical treatments, such as shampoos, conditioners, and follicular serums containing caffeine (which promotes scalp circulation), *Chrysanthemum indicum* (which enhances stem cell activation), and bitter melon (which alleviates follicular oxidative stress), optimize the microenvironment for follicular growth (52, 53). This dual approach of “oral internal regulation + external nourishment” creates a synergistic effect, improving the overall scalp environment and directly counteracting the negative effects of androgens on hair follicles, providing comprehensive hair regeneration support for AGA patients.

Pumpkin seed oil (PSO), with its plant sterols and essential fatty acids, has become a natural anti-androgen option (54). Its core ingredient, β-sitosterol, inhibits 5α-reductase to reduce DHT (55). A study of 76 male AGA patients showed that 400 mg of PSO taken daily for 24 weeks increased hair count by 40% (significantly higher than the placebo group’s 10%) without severe adverse reactions (21). Additionally, its fatty acids (linoleic acid, alpha-linolenic acid) and zinc improve scalp nutrition and reduce chronic inflammation, nourishing hair follicles and reducing androgen damage (54). It is particularly suitable for patients who cannot tolerate synthetic anti-androgen drugs. In this network meta-analysis, PSO showed statistically significant improvement in blind doctor evaluations of hair growth.

### 4.4 Limitations

First, the number of included studies and sample sizes were limited. We only identified 20 eligible RCTs, and some supplements had only 1–2 small-scale studies supporting them, which restricted the generalization of the results. The insufficient sample size also led to some comparisons lacking sufficient statistical power, such as the terminal-to-vellus hair ratio, where effect sizes were small and highly variable across studies, and no significant differences were observed even in combined analyses.

Second, there was considerable heterogeneity among the interventions. The types of dietary supplements included were diverse, with varying ingredient compositions (e.g., single plant extracts vs. multi-component vitamins and minerals). There were also differences in dosage, frequency of administration, and treatment duration across studies. Although heterogeneity was partially addressed by using random-effects models, it may still have affected the precision of the effect size estimates.

Moreover, publication bias was evident, and safety data reporting was insufficient. Funnel plot tests for hair density and blind doctor evaluations showed asymmetry, suggesting the potential for publication bias or small sample effects. Additionally, most studies focused on short-term safety (adverse events during the intervention period), resulting in a lack of long-term safety data, which limits the ability to assess the safety of prolonged dietary supplement use.

Finally, this study did not stratify or directly compare dose differences across dietary supplements. Because formulations and dosages varied substantially among studies, and some trials did not report detailed active-ingredient content or exact administered doses, it was not feasible to construct a robust dose-stratification model. Consequently, our findings reflect the relative effectiveness of each supplement at the doses specified within its respective trial, rather than head-to-head comparability under dose-equivalent conditions.

### 4.5 Clinical implications and recommendations

The potential of dietary supplements for treating AGA holds significant clinical and practical value: on the one hand, this approach aligns with the increasing consumer demand for natural and gentle interventions; on the other hand, as adverse events associated with conventional treatments like finasteride become more apparent, exploring safe and effective alternatives or adjunctive solutions further emphasizes the importance of this research. It should be emphasized that the current body of research remains at an exploratory stage; all conclusions below are derived from limited existing evidence and require confirmation by further high-quality studies. Based on the potential effect trends observed to date: if the primary focus is increasing hair quantity (hair density), preliminary results indicate that Nutrafol shows a positive signal on this outcome, with AMSbzs, AMS, and tocotrienol serving as potential alternatives when tolerability is suboptimal; if the goal is to reverse follicular miniaturization and increase the proportion of terminal hairs (terminal hair density), ALRV5XR demonstrates a preliminary favorable trend, with additional benefit potentially achieved by adding Nutrafol or probiotics when necessary; if the emphasis is on visible and tactile improvements in hair appearance and feel, pumpkin seed oil (PSO) may be considered as an option. The subgroup analysis of control schemes shows that dietary supplements do offer visible clinical benefits over placebo, but their overall effect does not surpass first-line prescription drugs. Clinically, they should be used as adjuncts to standard treatments, with a preference for combining them with conventional treatments to maximize visible effects. The female age subgroup suggests that postmenopausal women are the primary beneficiaries of nutritional supplements. The gender subgroup suggests that women show more stable benefits in terminal hair density, while the evidence for men is insufficient, and supplements should be considered as adjunctive therapy. It is recommended to combine dietary supplements with topical active agents (such as shampoos, conditioners, and follicular serums containing ingredients similar to those in oral supplements) when there are no contraindications to achieve synergistic effects. In practice, selecting 1–2 core interventions at a time is advised to avoid excessive combinations that could lead to adherence or adverse reaction issues.

## 5 Conclusion

This study, through a network meta-analysis, systematically evaluated the efficacy and safety of dietary supplements for treating AGA. Results indicated that multiple dietary supplements exhibited positive signals in improving hair-related outcomes: Nutrafol performed best for increasing hair density, ALRV5XR ranked highest for terminal hair density, and pumpkin seed oil (PSO) showed a high SUCRA probability in blinded physician-rated outcomes. These findings suggest that different formulations may influence hair growth through multi-target mechanisms (e.g., modulation of androgen metabolism, attenuation of oxidative stress, and improvement of follicular microcirculation).

With respect to safety, all supplements were generally well tolerated. Nevertheless, these results should be interpreted with caution. Given the variation across included studies in formulation composition, dosing, treatment duration, and concomitant use of topical active agents, the current evidence is not yet sufficient to support direct clinical recommendations or regimen selection. Future research should comprise high-quality randomized controlled trials with larger sample sizes and longer follow-up, and incorporate stratified analyses by sex, age, and special populations (e.g., pregnant individuals, patients with renal impairment) to further validate efficacy and long-term safety, thereby providing more robust evidence for the standardized clinical use of dietary supplements in AGA.

## Data Availability

The data used to support the findings of this study are available from the published literature.
