# Supplementary material for: Effects of dietary supplements on androgenetic alopecia: a systematic review and network meta-analysis
Source: Front Nutr. 2026 Jan 5;12:1719711. doi: 10.3389/fnut.2025.1719711 (PMC12812558; doi:10.3389/fnut.2025.1719711)
Supplement: Supplementary file 1 [file Table_1.docx]

Supplementary Table S1 The categories of supplements and their potential mechanisms of action

| **Category** | **Supplement** | **Mode of action** |
| --- | --- | --- |
| Androgen modulators | Lipidosterolic extract of Serenoa repens and betasitostero | Inhibit 5α-reductase, reduce DHT, anti-inflammatory |
|  | Serenoa repens | Inhibit 5α-reductase |
|  | pumpkin seed oil（PSO） | Inhibit 5α-reductase, anti-inflammatory, improve microcirculation |
|  | FSE-M | Inhibit 5α-reductase, reduce DHT, antioxidant, enhance local blood flow |
| Antioxidants | tocotrienol | Scavenge ROS, inhibit lipid peroxidation |
|  | omega 3&6 | Anti-inflammatory, improve microcirculation, decrease pro-inflammatory mediators |
|  | CT-LJ | Antioxidant, promote microcirculation, modulate nutrient metabolism |
| Probiotics | Probiotics | Improve gut microbiota, modulate systemic metabolism, optimize the follicular microenvironment |
| Growth hormone regulators | Capsaicin and isoflavone | Upregulate IGF-I expression and secretion, regulate the hair-follicle cycle |
| Marine protein-based supplements | Viviscal | Promote collagen and antioxidation, support keratinization and metabolism, prolong anagen |
| Multi-ingredient supplements | Nutrafol | Anti-inflammatory, antioxidant, suppress DHT |
|  | AMSbzs、AMS | Alleviate oxidative stress and inflammation, promote hair epithelial cell growth |
|  | PPT5α | Inhibit 5α-reductase, anti-inflammatory, antioxidant, provide substrates for keratin synthesis, strengthen the hair shaft |
|  | ALRV5XR | Inhibit 5α-reductase, anti-inflammatory, antioxidant, promote hair collagen synthesis |
|  | Lambdapil | Inhibit 5α-reductase, promote keratin synthesis |
|  | AGA-P | Inhibit 5α-reductase, support keratin synthesis, antioxidant |

Supplementary Table S2 Basic information of all the papers

| study | sample size（Treatment group/Other groups) | sex（M/F） | mean age（Treatment group/Other groups) | intervention | participants | treatment time(weeks) | outcome | randomization method |
| --- | --- | --- | --- | --- | --- | --- | --- | --- |
| Narda 2017 | 23/12 | 35/0 | 40.6±2.5/46.4±2.5 | Lambdapil:L-Cysteine（2000mg/d）+Serenoa repens（200mg/d）+scouring rush（14.28mg/d）+zinc（20mg/d) etc；placebo | AGA | 24w | ③ | Random |
| Feldman 2021 | 23/23 | 46/0 | 48.3±7.9/47.5±12.9 | ALRV5XR:plant extracts+vitamins+minerals(1684mg/d)+shampoo（external use）+conditioner（external use）+Hair follicle serum（external use）；placebo | AGA | 24w | ②④ | Random Number Table |
| García-N 2024 | 67/69 | 62/74 | 32.6±4.8/33.1±5.2 | Probiotics:one capsules/d；placebo | AGA | 16w | ①②④ | Blocked Randomization |
| Moers-C 2011 | 39/17 | 26/30 | 43.4±9.7/44.9±8.6 | FSE-M:fenugreek seed extract（300mg/d）+Vitamin C(120mg/d)+ Vitamin E(20mg/d)+ Niacinamide(18mg/d)+Pantothenic Acid(6mg/d)+Zinc(5mg/d) etc；placebo | AGA | 24w | ①② | Random Number Table |
| Milani 2025 | 106/119 | 165/60 | 39±14/41±14 | AGA-P:Serenoa repens（320 mg/d）+Cucurbita pepo（320 mg/d）+L-Cystine（425 mg/d）+Vitamin C+Zinc+CT；CT | AGA | 24w | ③ | Random |
| Beoy 2010 | 21/17 | 36/2 | NI | Tocotrienol:100mg/d；placebo | NA | 32w | ① | Random |
| Cho 2014 | 37/39 | 76/0 | NI | PSO:400 mg/d；placebo | AGA | 24w | ①③ | Random Number Table |
| ROSSI 2012 | 50/50 | 100/0 | NI | ESR:320mg/d；finasteride 1mg/d | AGA | 24w | ③ | Random |
| Harada 2007 | 31/17 | 25/23 | NI | CI:capsaicin（6mg/d）+isoflavone（75mg/d）；placebo | 70.8%AGA;29.2%AA | 20w | ③ | Random |
| Ablon 2018 | 25/14 | 0/39 | 48.3±10.5/53.14±5.7 | Nutrafol Women’s:four capsules/d；placebo | AGA | 24w | ①②④ | Random |
| Prager 2002 | 13/13 | 26/0 | NI | ESR:liposterolic extract of serenoa repens（400mg/d）+β-sitosterol（100mg/d）；placebo | AGA | 21w | ③ | Random |
| Bhatia 2025 | 75/37 | 112/0 | 40.9±8/41.2±1.4 | Nutrafol:four capsules/d；placebo | AGA | 24w | ③ | Random |
| Arias 2023 | 24/23 | 0/47 | NI | PPT5α:plant extract(310mg/d)+zinc(10mg/d）+ amino acid（100mg/d）+ hydrolyzed collagen（250mg/d）；placebo | AGA | 24w | ③ | Random |
| De Biasio 2023 | 40/40 | 40/40 | 44.5±12.6/43.3±11.6 | AMS:apple extract（800 mg/d）；placebo | AGA | 24w | ② | Random Number Table |
| Ablon 2016 | 30/30 | 60/0 | 42.8±7.7/46.1±7.6 | Viviscal:Marine complex（500mg/d）；placebo | AGA | 24w | ① | Random |
| Ablon 2021 | 40/30 | 0/70 | 54.6±5.9/55.8±7.3 | Nutrafol Women’s Balance:four capsules/d；placebo | AGA | 24w | ①②③ | Random |
| Seok 2015 | 45/49 | 46/48 | 40.8±9.7/41.39±11.27 | MK-R7:extract of cistanche tubulosa(300mg/d)+extract of laminaria japonica(100mg/d);placebo | AGA | 16w | ① | stratified permuted block randomization |
| Tenore 2018 | 125/125 | 116/134 | 42.1±11.1/43.5±10.2 | AMSbzs:AMS(800mg/d）+biotin(0.40mg/d）+selenium(160μg/d)+zinc(42mg/d）;AMS:apple extract（800mg/d） | AGA | 16w | ① | Random Number Table |
| Le Floc’h 2015 | 80/40 | 0/120 | 42.3±/41.8±8.1 | Omega3&6:fish oil(460mg/d)+blackcurrant seed oil(460mg/d)+VE(5mg/d)+VC（30mg/d)+lycopene（1mg/d);NI | AGA | 24w | ③④ | Random Number Table |

FSE-M:fenugreek seed extract and microelement；PSO:pumpkin seed oil；ESR：extract of serenoa repens；CI：capsaicin and isoflavone；Nutrafol:standardized plant extracts（saw palmetto, maca, astaxanthin, curcumin, tocotrienols, ashwagandha）and micronutrient；Nutrafol Women’s Balance:Nutrafol+maca+astaxanthin；F:female；m:male；CT：conventional therapy；NI: No intervention；①Hair density；②Terminal hair density；③Blind assessment by doctors；④Final hair/soft fur

Supplementary Table S3 Results of network Meta-Analysis(Hair density)

| Nutrafol | Nutrafol |  |  |  |  |  |  |  |  |  |
| --- | --- | --- | --- | --- | --- | --- | --- | --- | --- | --- |
| AMSbzs | 0.05 (-0.62,0.73) | AMSbzs |  |  |  |  |  |  |  |  |
| tocotrienol | 0.05 (-0.77,0.87) | -0.01 (-0.88,0.87) | tocotrienol |  |  |  |  |  |  |  |
| AMS | 0.09 (-0.53,0.71) | 0.04 (-0.22,0.30) | 0.04 (-0.79,0.88) | AMS |  |  |  |  |  |  |
| PSO | 0.32 (-0.34,0.97) | 0.26 (-0.46,0.99) | 0.27 (-0.59,1.13) | 0.23 (-0.45,0.90) | PSO |  |  |  |  |  |
| MK-R7 | 0.33 (-0.27,0.92) | 0.27 (-0.40,0.94) | 0.28 (-0.54,1.10) | 0.24 (-0.38,0.85) | 0.01 (-0.64,0.66) | MK-R7 |  |  |  |  |
| Viviscal | 0.51 (-0.15,1.18) | 0.46 (-0.27,1.19) | 0.47 (-0.40,1.34) | 0.42 (-0.26,1.11) | 0.20 (-0.52,0.91) | 0.19 (-0.47,0.84) | Viviscal |  |  |  |
| FSE-M | 0.59 (-0.12,1.30) | 0.54 (-0.24,1.32) | 0.54 (-0.36,1.45) | 0.50 (-0.23,1.23) | 0.27 (-0.49,1.03) | 0.26 (-0.44,0.97) | 0.08 (-0.69,0.84) | FSE-M |  |  |
| Probiotics | **0.69 (0.13,1.25)** | 0.64 (-0.00,1.28) | 0.65 (-0.15,1.44) | **0.60 (0.02,1.19)** | 0.37 (-0.25,1.00) | 0.37 (-0.19,0.92) | 0.18 (-0.45,0.81) | 0.10 (-0.58,0.78) | Probiotics |  |
| placebo | **0.90 (0.48,1.33)** | **0.85 (0.33,1.38)** | **0.86 (0.16,1.56)** | **0.81 (0.36,1.27)** | **0.59 (0.09,1.09)** | **0.58 (0.17,0.99)** | 0.39 (-0.12,0.90) | 0.31 (-0.26,0.89) | 0.21 (-0.15,0.58) | placebo |
| **SMD，95% CI** | | | | | | | | | | |

expressed as MD and 95% CI. An MD greater than 0 indicates that longitudinal intervention measures outperform transverse intervention measures. A 95% CI value of 0 indicates that the difference is not statistically significant.

Supplementary Table S4 Results of network Meta-Analysis(Terminal hair density)

|  | _B_ |  |  |  |  |  |  |
| --- | --- | --- | --- | --- | --- | --- | --- |
| ALRV5XR | ALRV5XR |  |  |  |  |  |  |
| Nutrafol | 0.73 (-0.13,1.60) | Nutrafol |  |  |  |  |  |
| Probiotics | **1.17 (0.33,2.02)** | 0.44 (-0.12,1.00) | Probiotics |  |  |  |  |
| Viviscal | **1.24 (0.33,2.16)** | 0.51 (-0.15,1.17) | 0.07 (-0.56,0.70) | Viviscal |  |  |  |
| FSE-M | **1.28 (0.33,2.23)** | 0.55 (-0.16,1.26) | 0.11 (-0.57,0.79) | 0.04 (-0.73,0.81) | FSE-M |  |  |
| PPT5α | **1.57 (0.61,2.54)** | **0.84 (0.12,1.57)** | 0.40 (-0.30,1.10) | 0.33 (-0.45,1.11) | 0.29 (-0.53,1.12) | PPT5α |  |
| placebo | **1.58 (0.82,2.34)** | **0.85 (0.43,1.27)** | **0.41 (0.04,0.78)** | 0.34 (-0.17,0.85) | 0.30 (-0.27,0.87) | 0.01 (-0.58,0.60) | placebo |
| SMD，95% CI | | | | | | | |

expressed as MD and 95% CI. An MD greater than 0 indicates that longitudinal intervention measures outperform transverse intervention measures. A 95% CI value of 0 indicates that the difference is not statistically significant.

Supplementary Table S5 Results of network Meta-Analysis(Blind assessment by doctors)

| finasteride | finasteride |  |  |  |  |  |  |  |  |
| --- | --- | --- | --- | --- | --- | --- | --- | --- | --- |
| PSO | 0.54 (-2.26,3.34) | PSO |  |  |  |  |  |  |  |
| CI | 0.53 (-2.19,3.25) | -0.01 (-2.13,2.11) | CI |  |  |  |  |  |  |
| ESR | **0.58 (-0.39,1.55)** | 0.04 (-2.59,2.67) | 0.05 (-2.49,2.60) | ESR |  |  |  |  |  |
| Lambdapil | 1.31 (-1.38,3.99) | 0.76 (-1.31,2.83) | 0.78 (-1.19,2.74) | 0.72 (-1.78,3.23) | Lambdapil |  |  |  |  |
| omega3&6 | 1.48 (-1.06,4.01) | **0.93 (-0.94,2.81)** | **0.95 (-0.81,2.71)** | 0.90 (-1.45,3.24) | 0.17 (-1.53,1.87) | omega3&6 |  |  |  |
| Nutrafol | **1.53 (-0.91,3.97)** | **0.99 (-0.75,2.73)** | **1.00 (-0.62,2.62)** | 0.95 (-1.29,3.19) | 0.22 (-1.32,1.77) | 0.05 (-1.22,1.33) | Nutrafol |  |  |
| AGA-P | **1.85 (-0.66,4.36)** | **1.31 (-0.53,3.15)** | **1.32 (-0.40,3.05)** | 1.27 (-1.05,3.59) | 0.55 (-1.11,2.21) | 0.38 (-1.03,1.79) | **0.32 (-0.91,1.55)** | AGA-P |  |
| placebo | **2.27 (-0.05,4.58)** | **1.72 (0.15,3.29)** | **1.73 (0.31,3.16)** | **1.68 (-0.42,3.79)** | **0.96 (-0.39,2.31)** | **0.79 (-0.24,1.81)** | **0.73 (-0.02,1.49)** | **0.41 (-0.56,1.38)** | placebo |
| **SMD，95% CI** | | | | | | | | | |

expressed as MD and 95% CI. An MD greater than 0 indicates that longitudinal intervention measures outperform transverse intervention measures.

A 95% CI value of 0 indicates that the difference is not statistically significant.

Supplementary Table S6 Results of network Meta-Analysis(Final hair/soft fur)

| ALRV5XR | ALRV5XR |  |  |  |  |
| --- | --- | --- | --- | --- | --- |
| Probiotics | 0.18 (-0.53,0.90) | Probiotics |  |  |  |
| omega3&6 | 0.44 (-0.33,1.20) | 0.26 (-0.21,0.72) | omega3&6 |  |  |
| placebo | 0.45 (-0.22,1.11) | 0.26 (0.00,0.52) | 0.01 (-0.37,0.39) | placebo |  |
| Nutrafol | **1.04 (0.10,1.98)** | **0.86 (0.14,1.57)** | 0.60 (-0.17,1.37) | 0.59 (-0.08,1.26) | Nutrafol |
| SMD，95% CI | | | | | |

expressed as MD and 95% CI. An MD greater than 0 indicates that longitudinal intervention measures outperform transverse

intervention measures. A 95% CI value of 0 indicates that the difference is not statistically significant.

Supplementary Table S7 Meta-regression results

| Outcomes | Variable | P Value |
| --- | --- | --- |
| Hair density | Crowd | 0.683 |
|  | Gender | 0.945 |
|  | Time | 0.123 |
|  | Types of supplements | 0.772 |
| Terminal hair density | Gender | 0.578 |
|  | Time | 0.793 |
|  | Intervening measure | 0.065 |
| Blinded physician evaluations | Time | 0.567 |
|  | Gender | 0.548 |
|  | Scales | 0.962 |
|  | Comparison | 0.049 |

Figure S1 Risk of bias assessment included in the study.


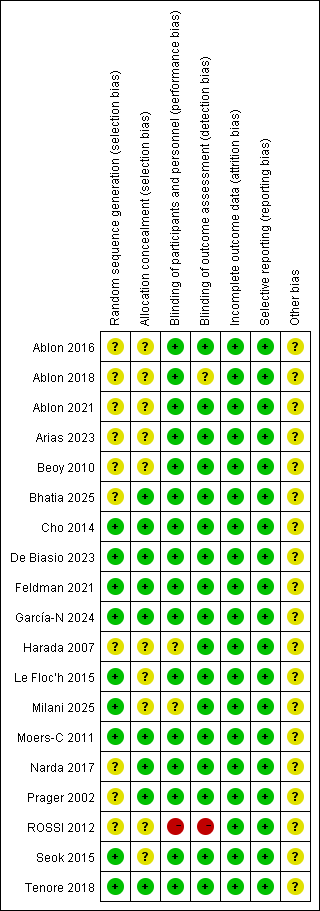


FigureS2 Meta analysis of the Hair density


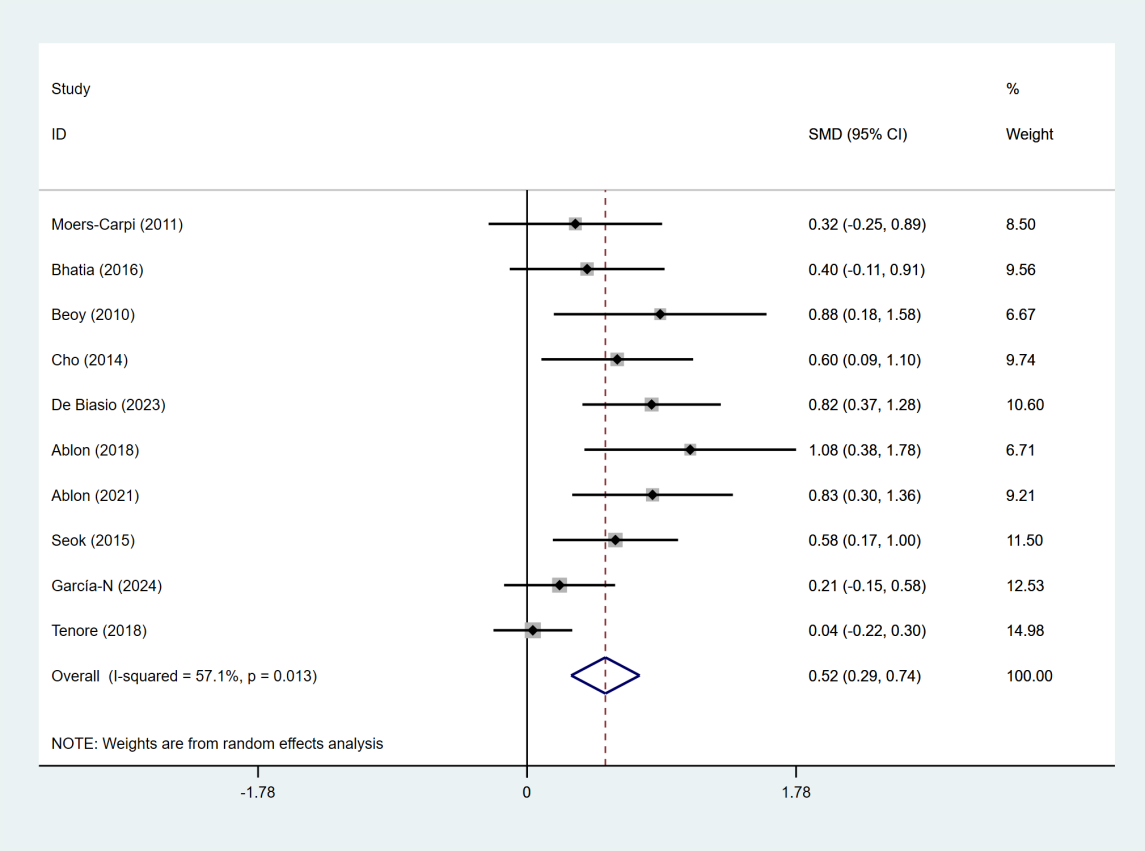


Figure S3 Meta-analysis of different gender in hair density studies

**
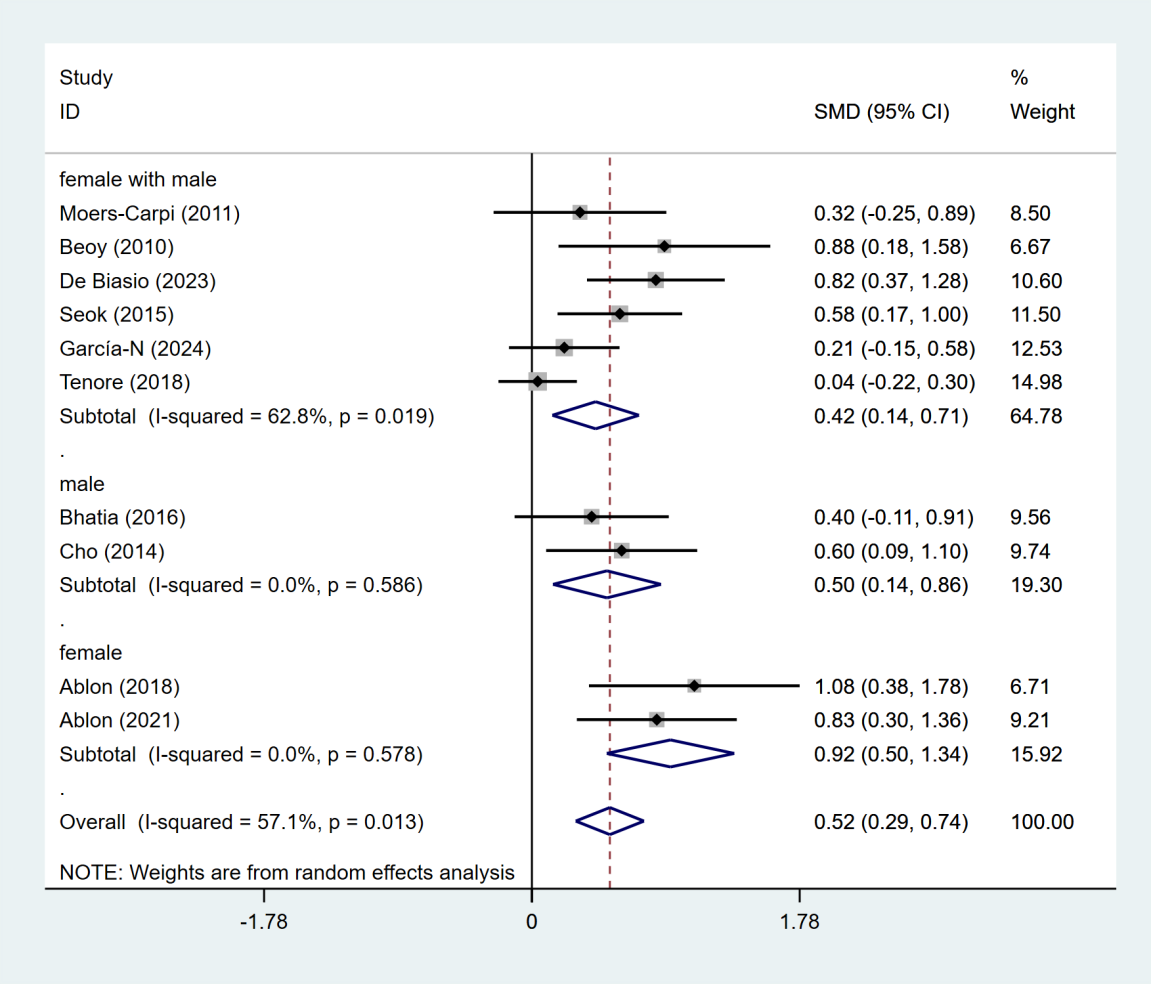
**

Figure S4 Meta-analysis of different intervention time in hair density studies

**
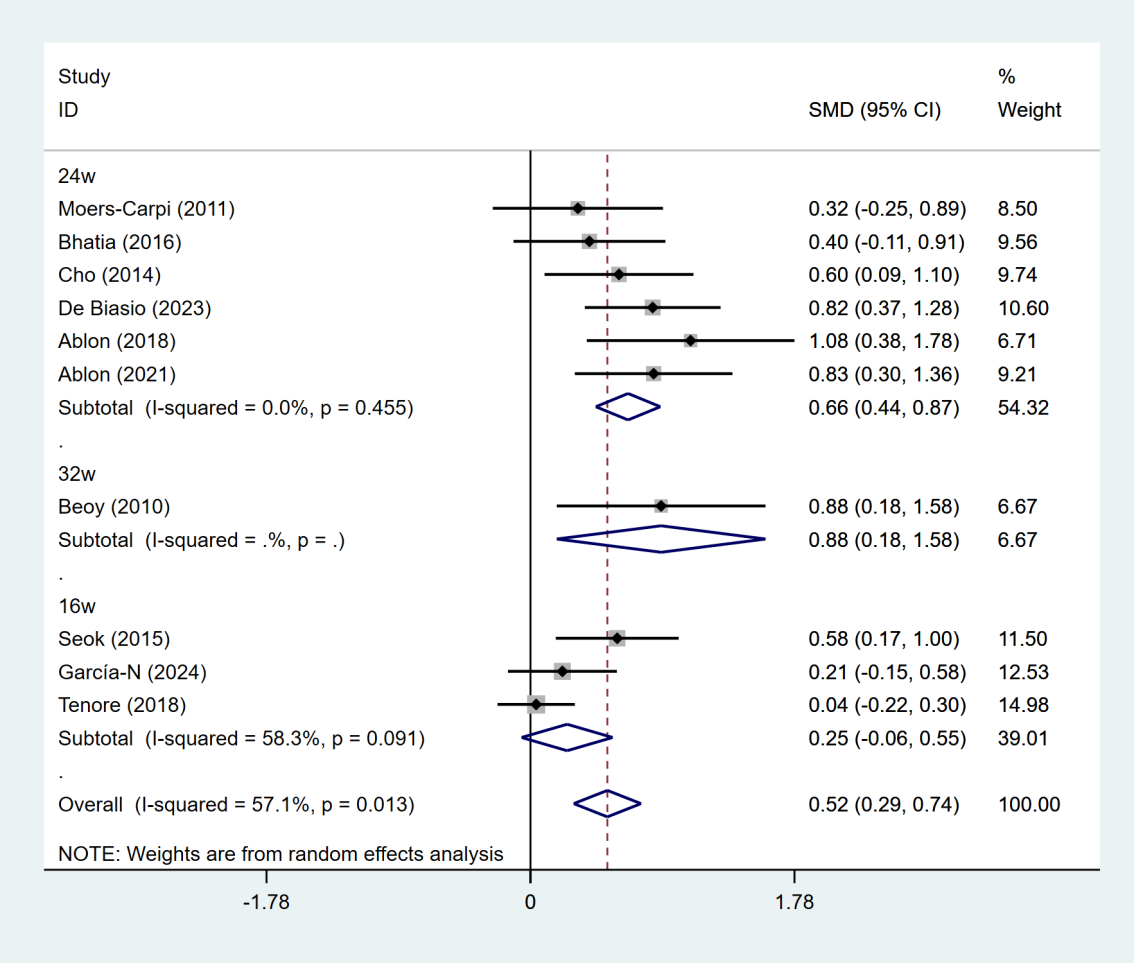
**

Figure S5 Meta-analysis of different types of dietary supplements in hair-density studies

**
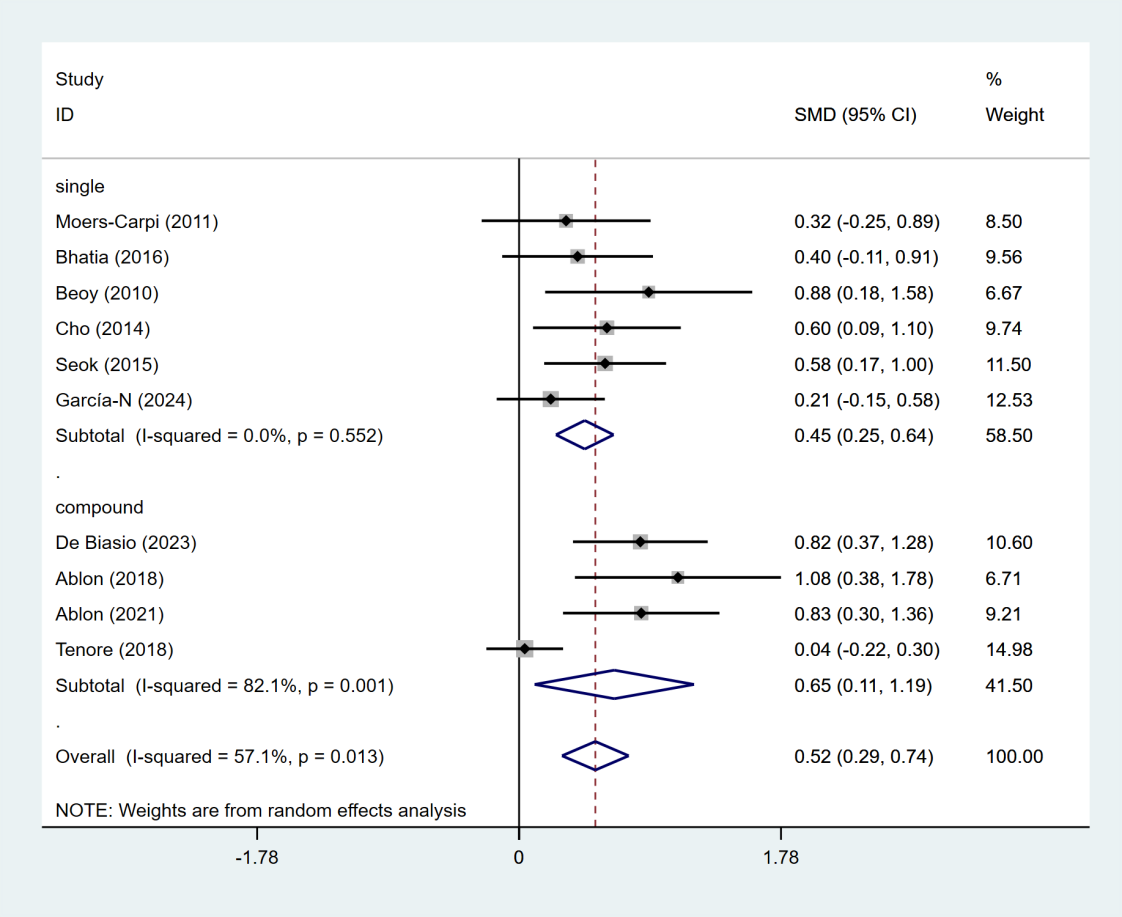
**

Figure S6 A sensitivity analysis on the Hair density


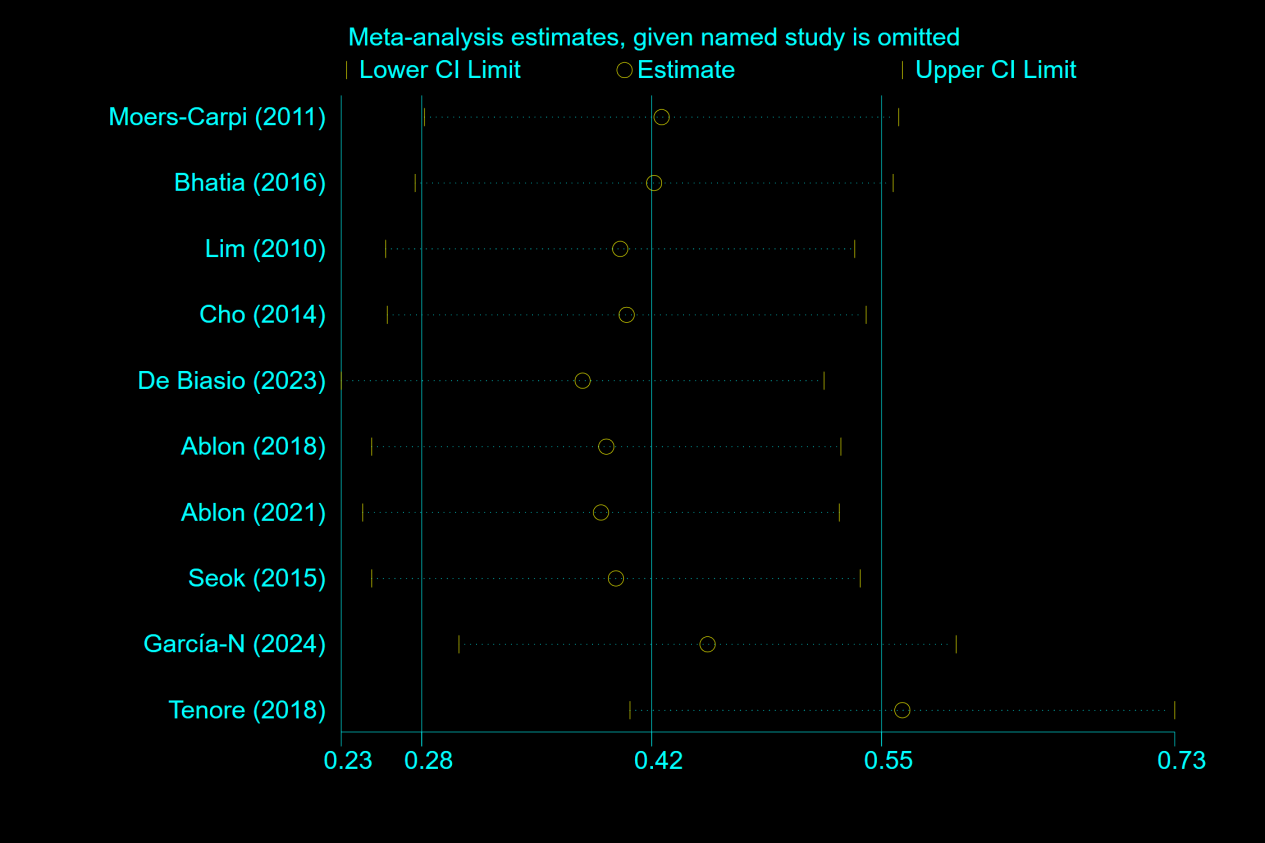


Figure S7 Meta analysis of Terminal hair density


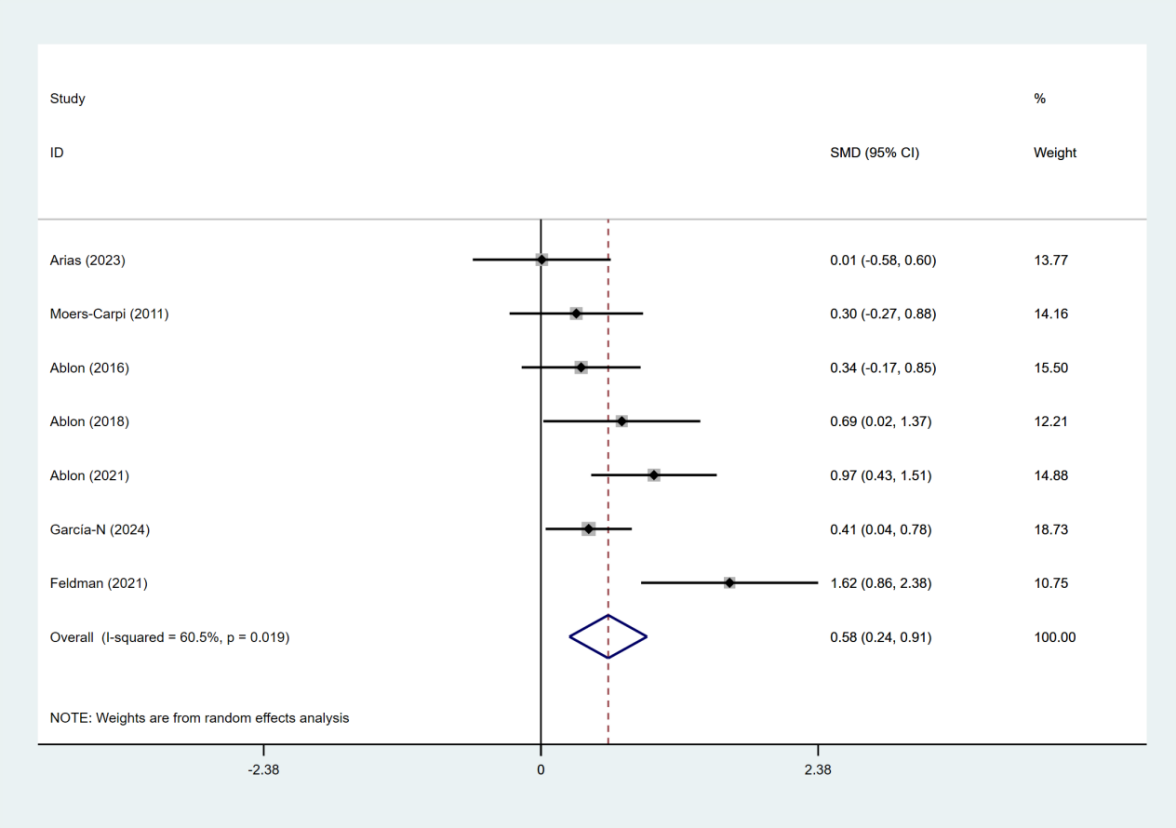


Figure S8 Meta-analysis of different gender in Terminal hair density studies


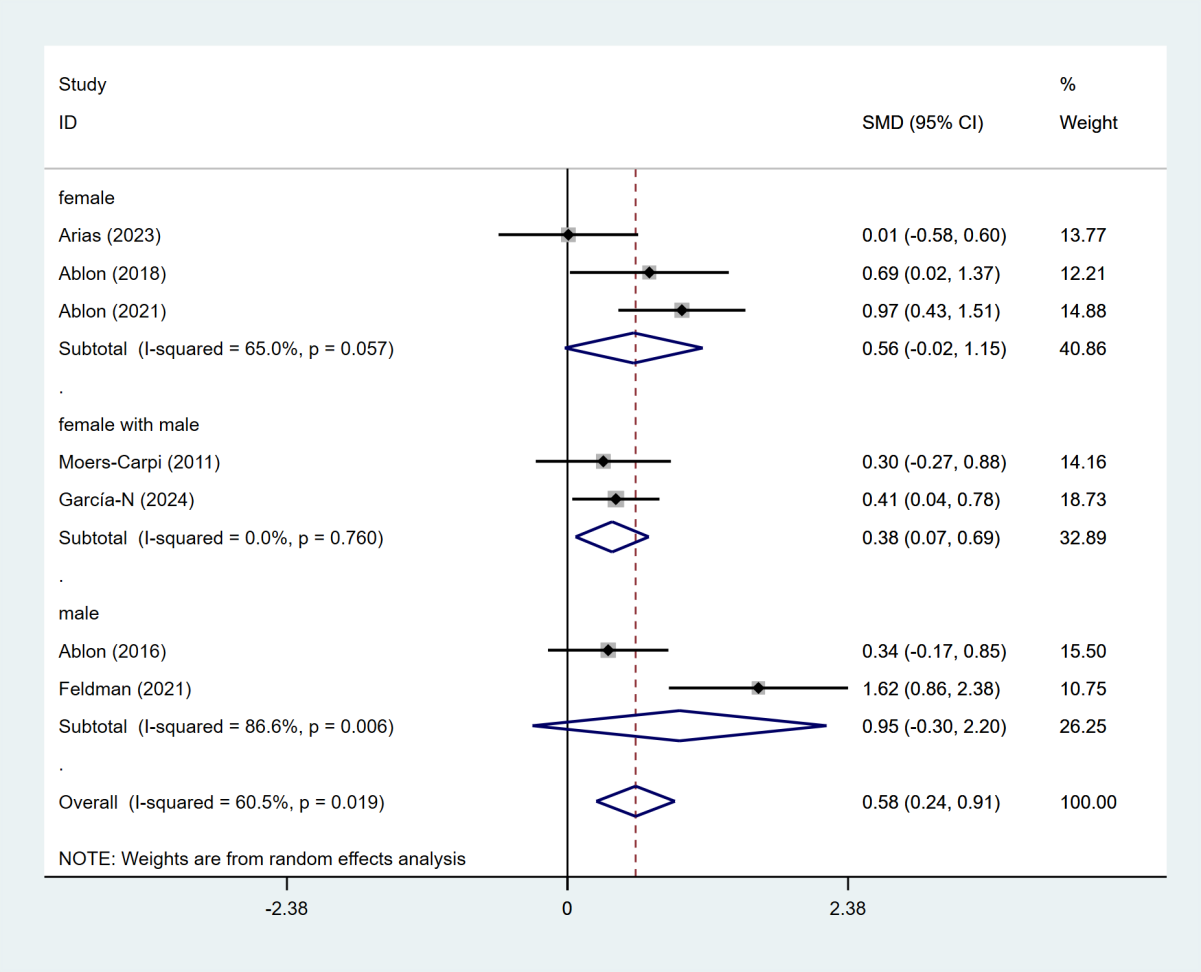


Figure S9 Meta-analysis of different intervention time in Terminal hair density studies


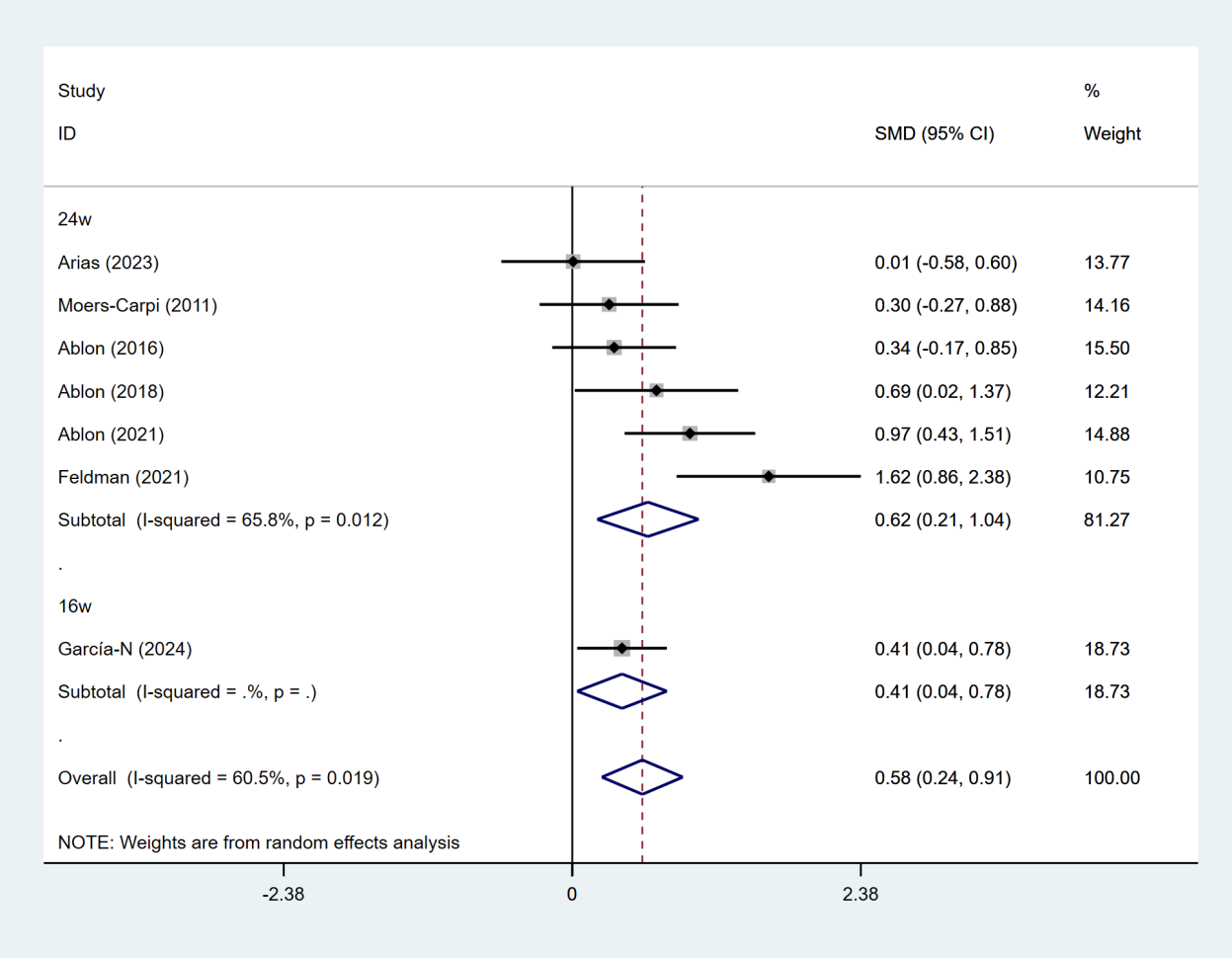


Figure S10 Meta-analysis of different intervention measures in Terminal hair density studies


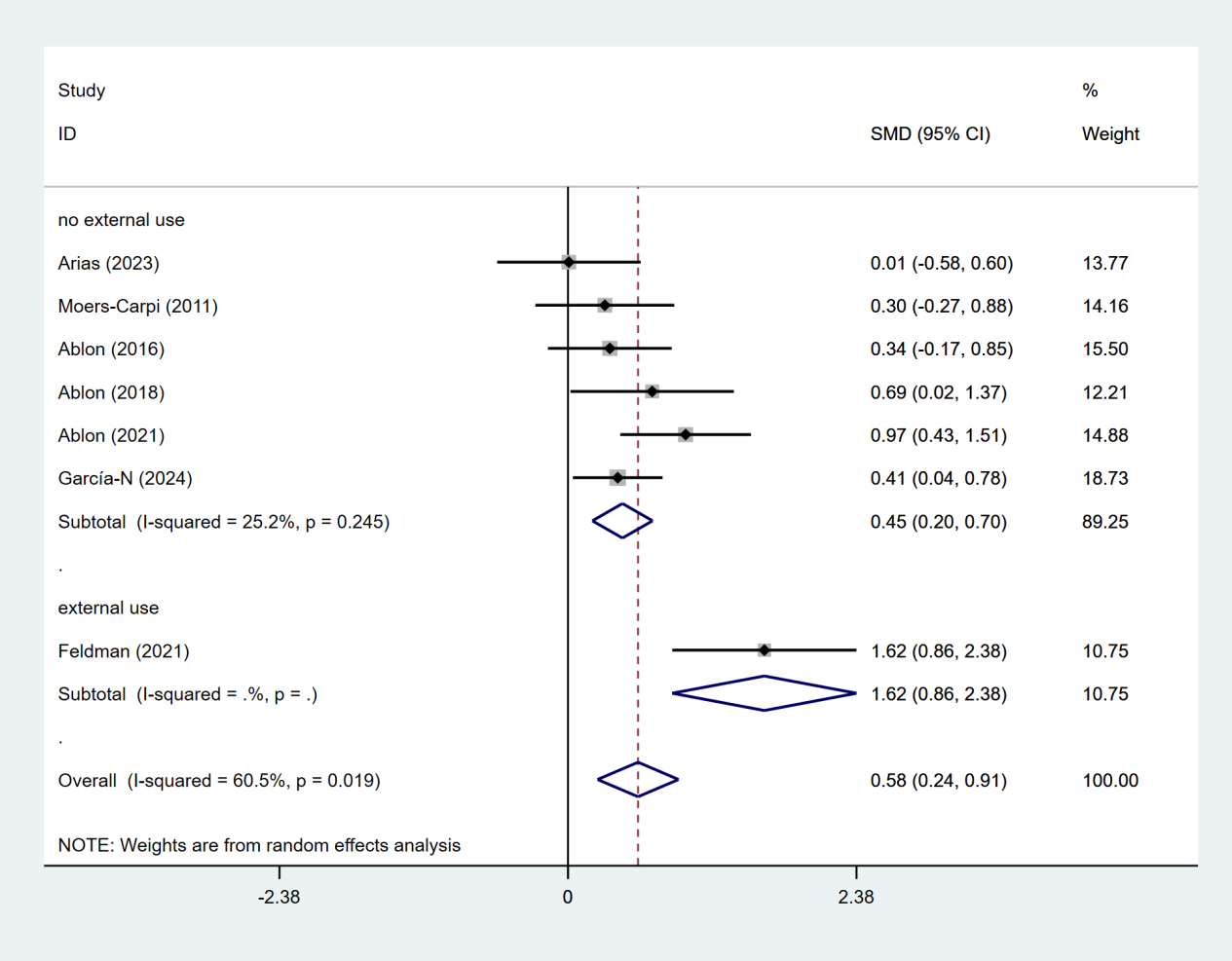


Figure S11 A sensitivity analysis on the Terminal hair


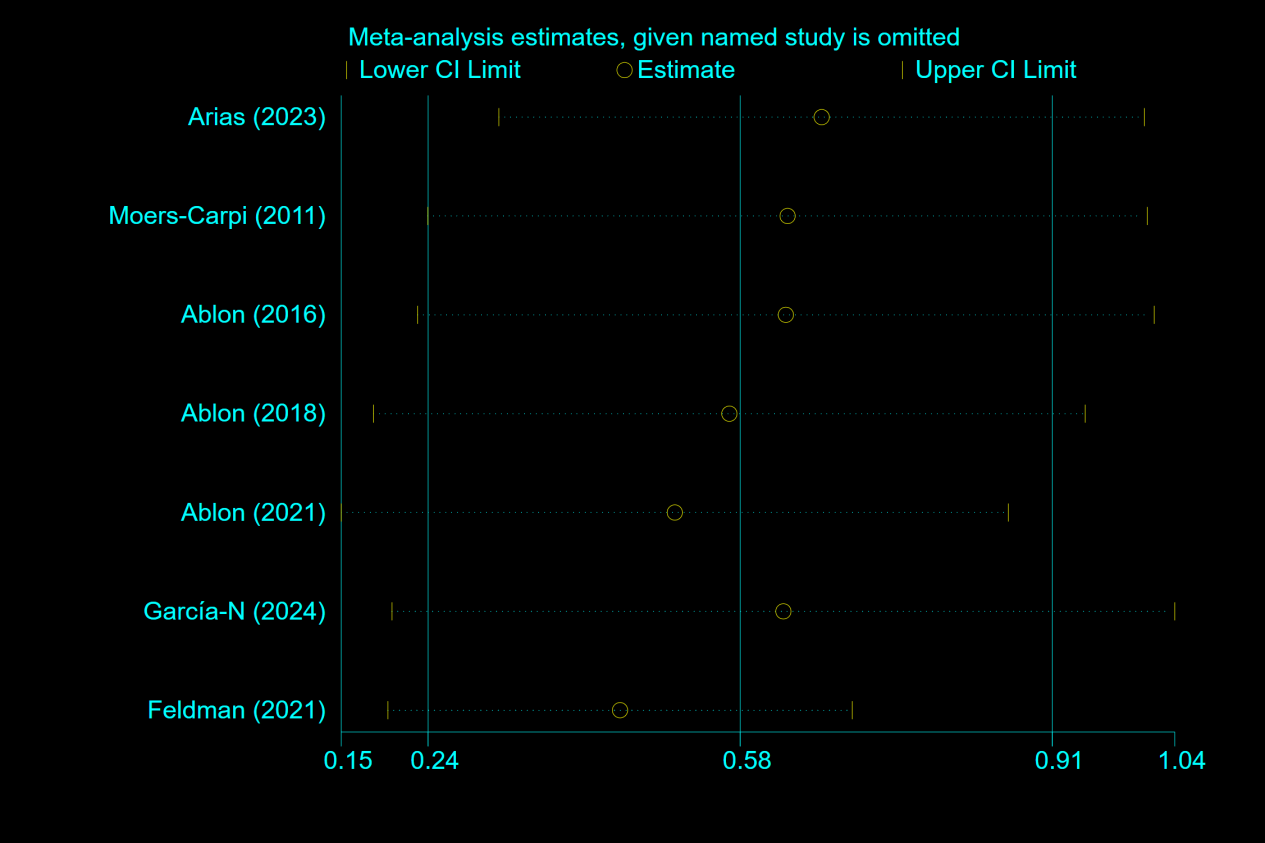


Figure S12 Meta analysis of Blind assessment by doctors


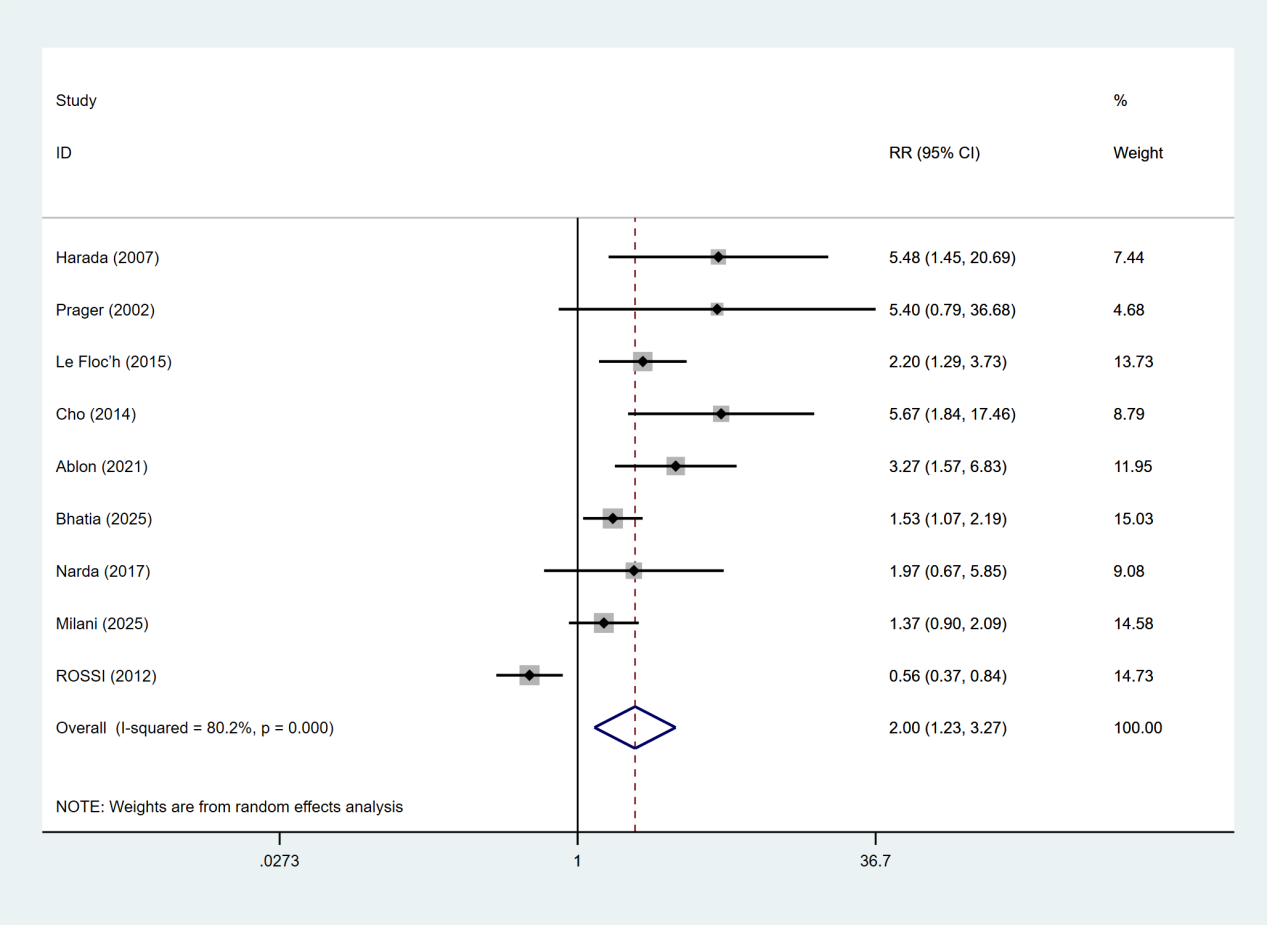


Figure S13 A sensitivity analysis on the Blind assessment by doctors


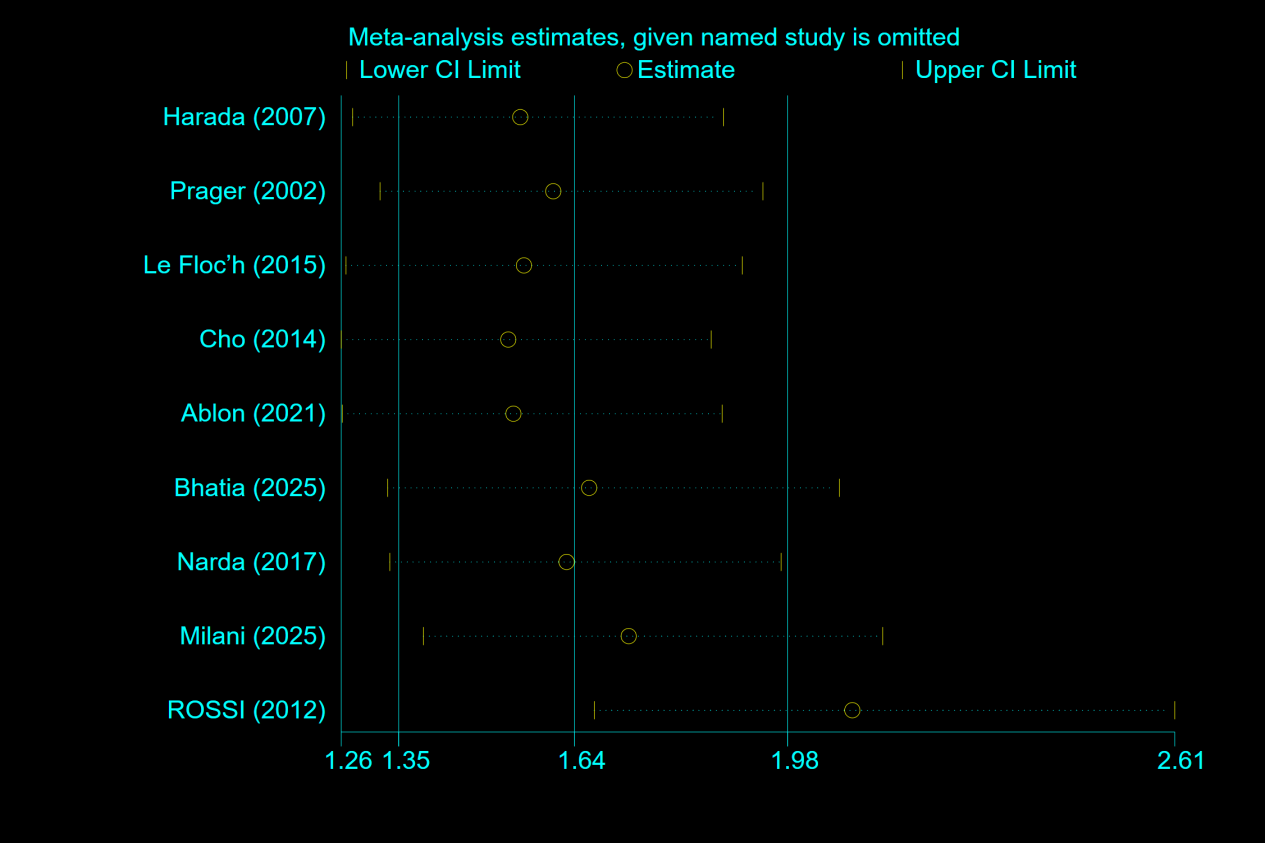


Figure S14 Meta-analysis of different control schemes in blinded physician evaluations


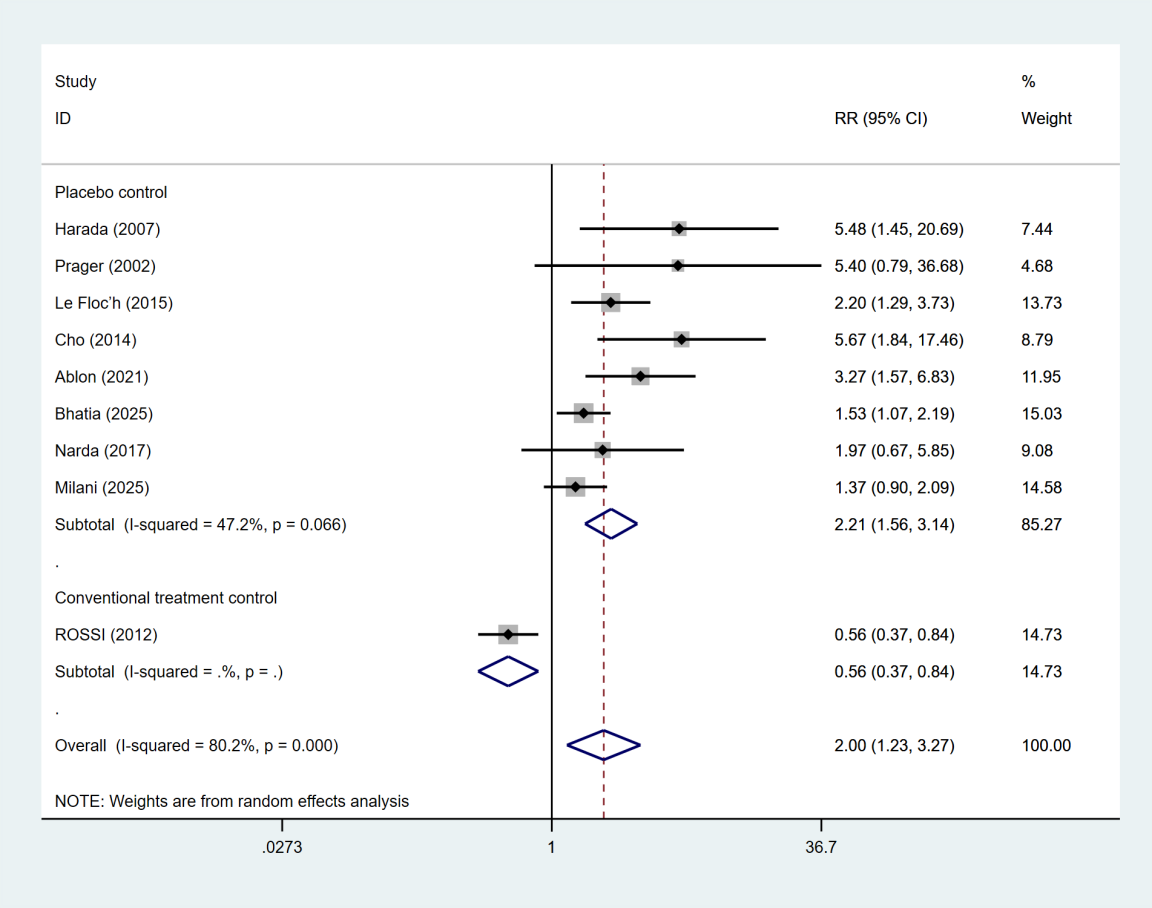


Figure S15 Meta analysis of different gender in blinded physician evaluations


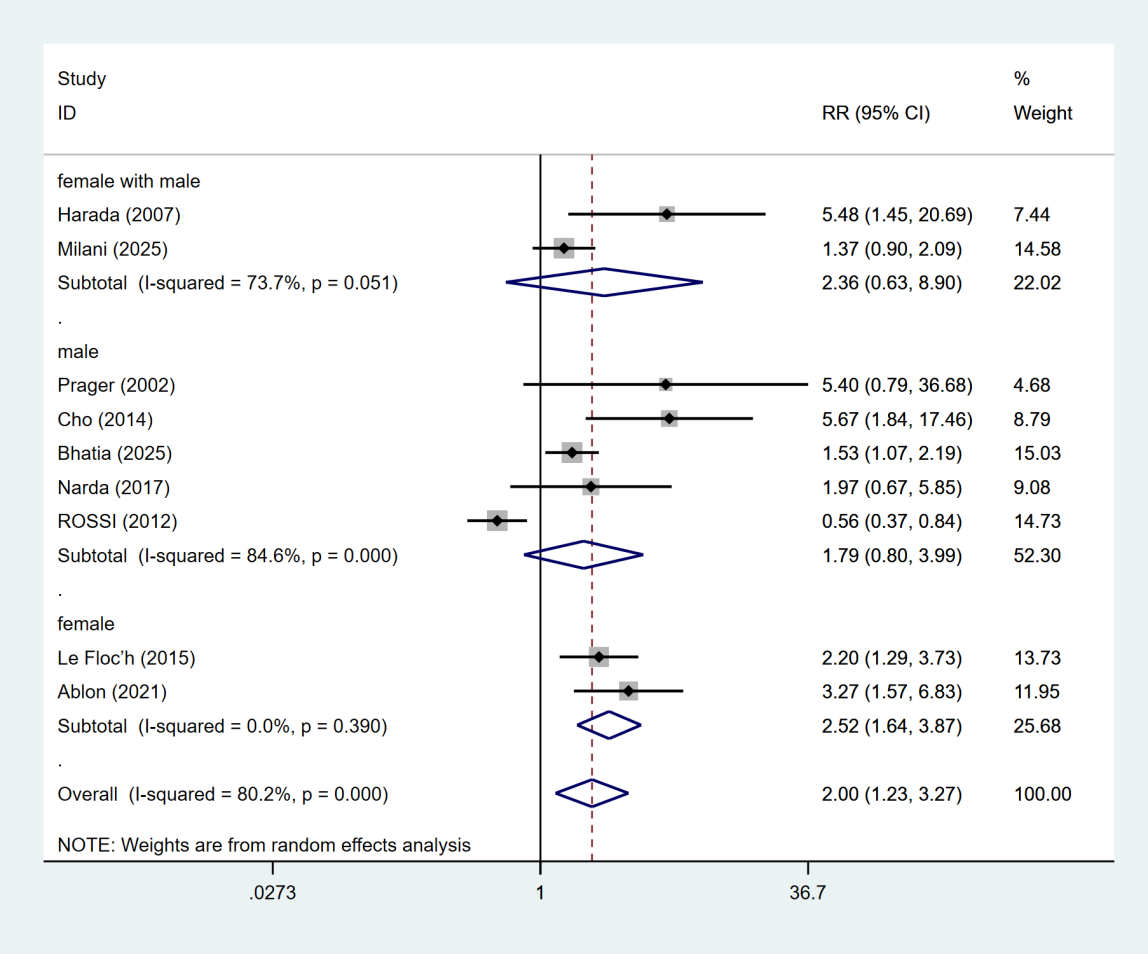


Figure S16 Meta analysis of Ratio of terminal hair to bristle hair


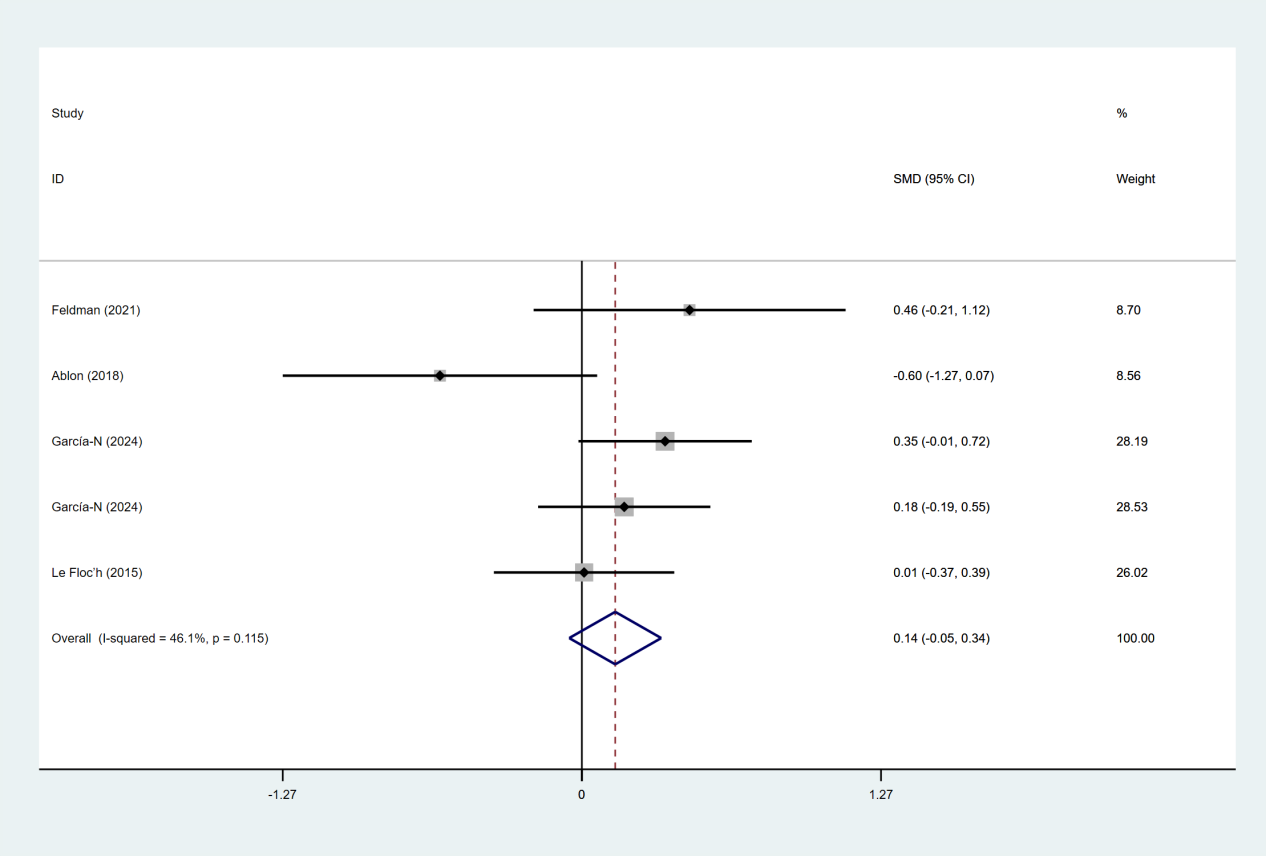


Figure S17 Meta analysis of dietary supplement versus placebo


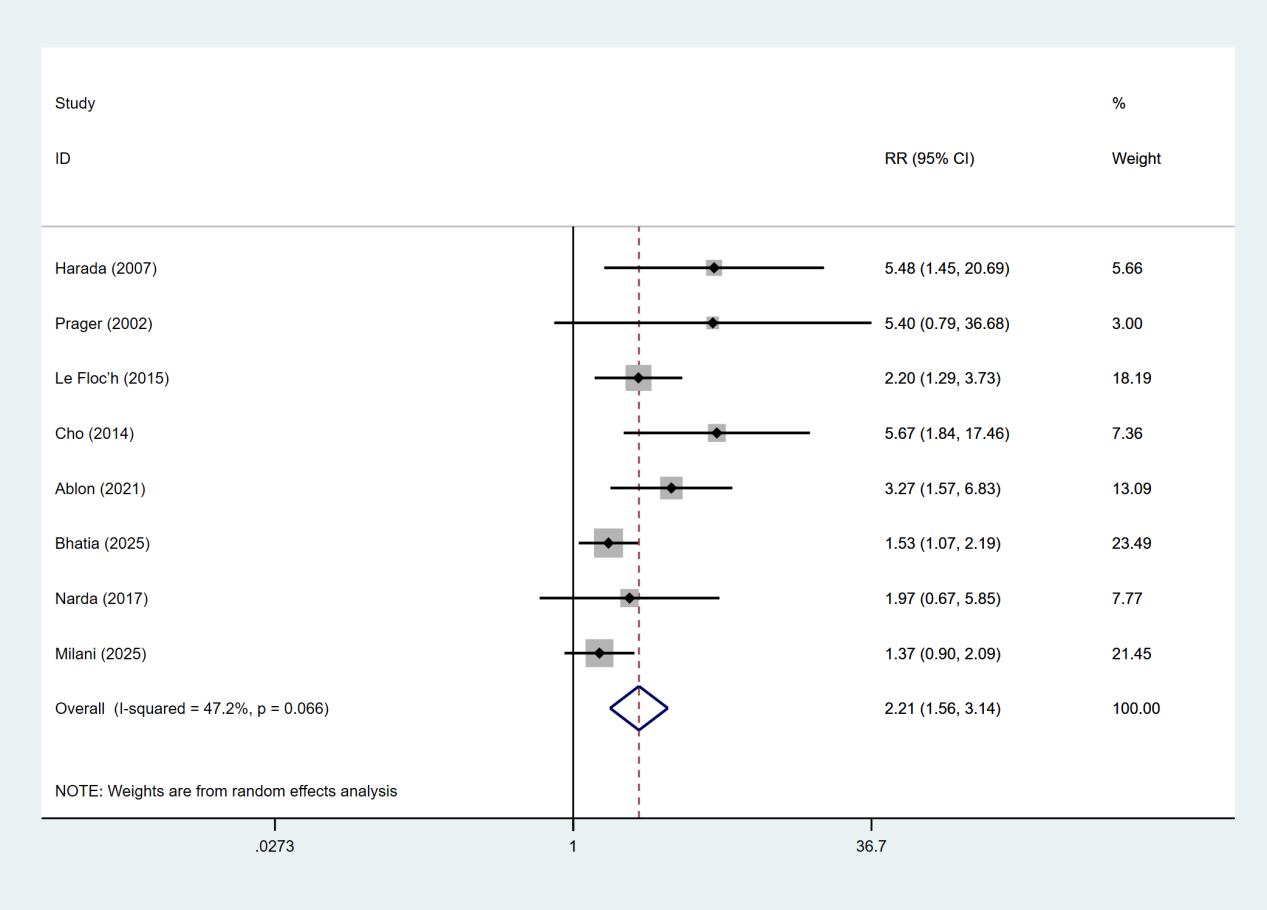


Figure S18 Meta analysis of dietary supplement versus conventional therapy


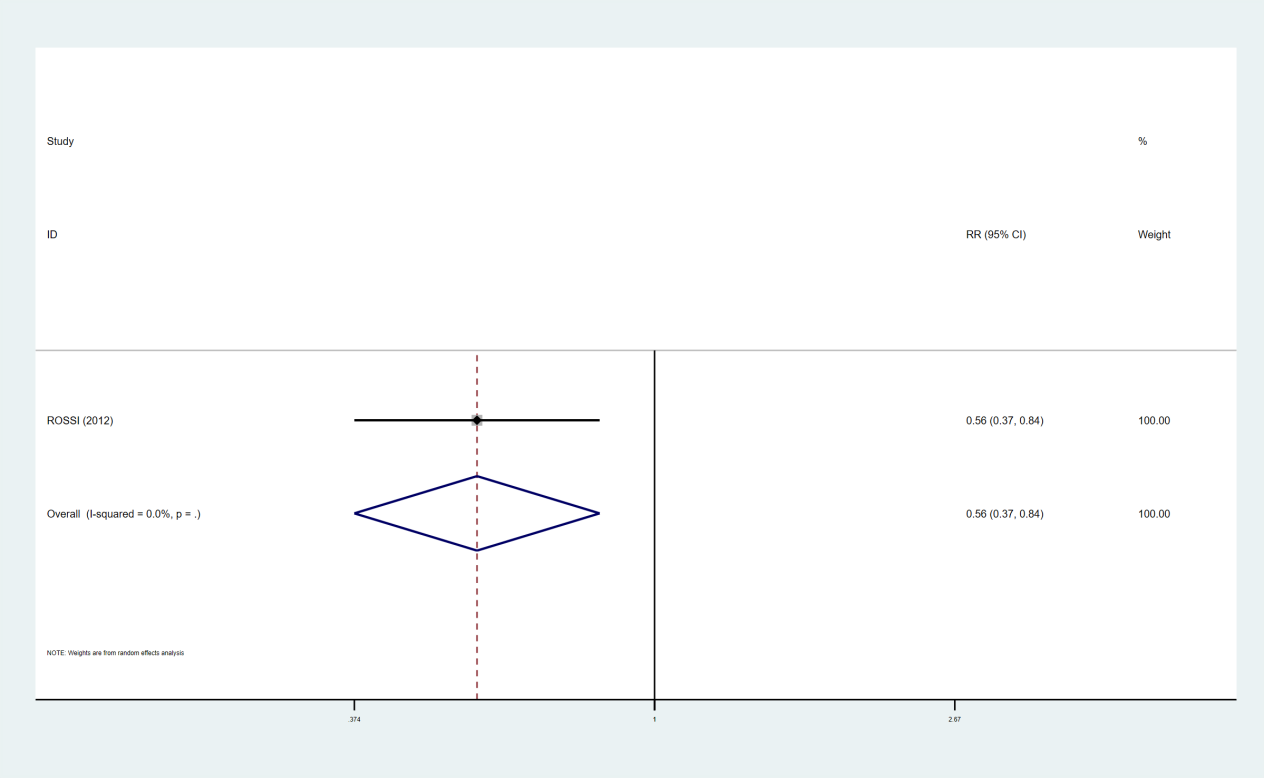


Figure S19 Meta analysis of menopausal subgroup


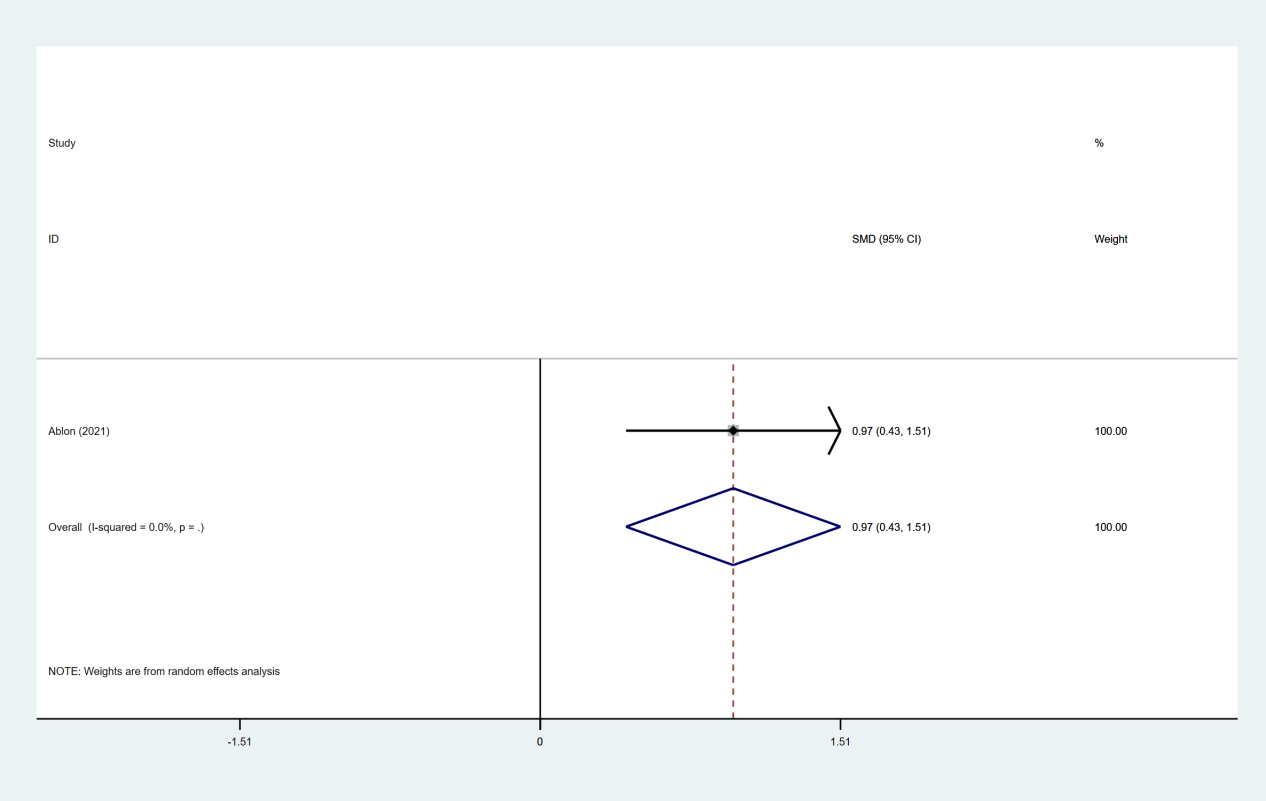


Figure S20 Meta analysis of Non-menopausal subgroup


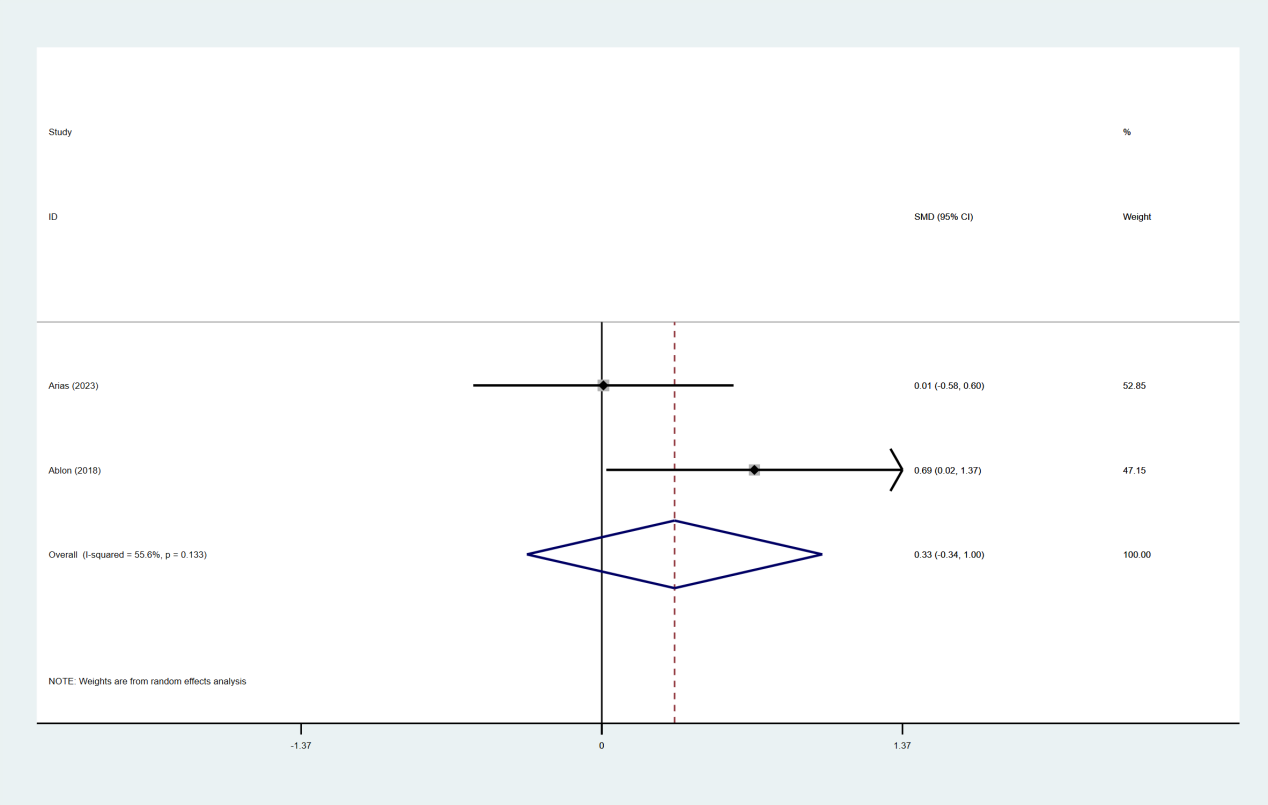


Figure S21 Meta analysis of Female subgroup


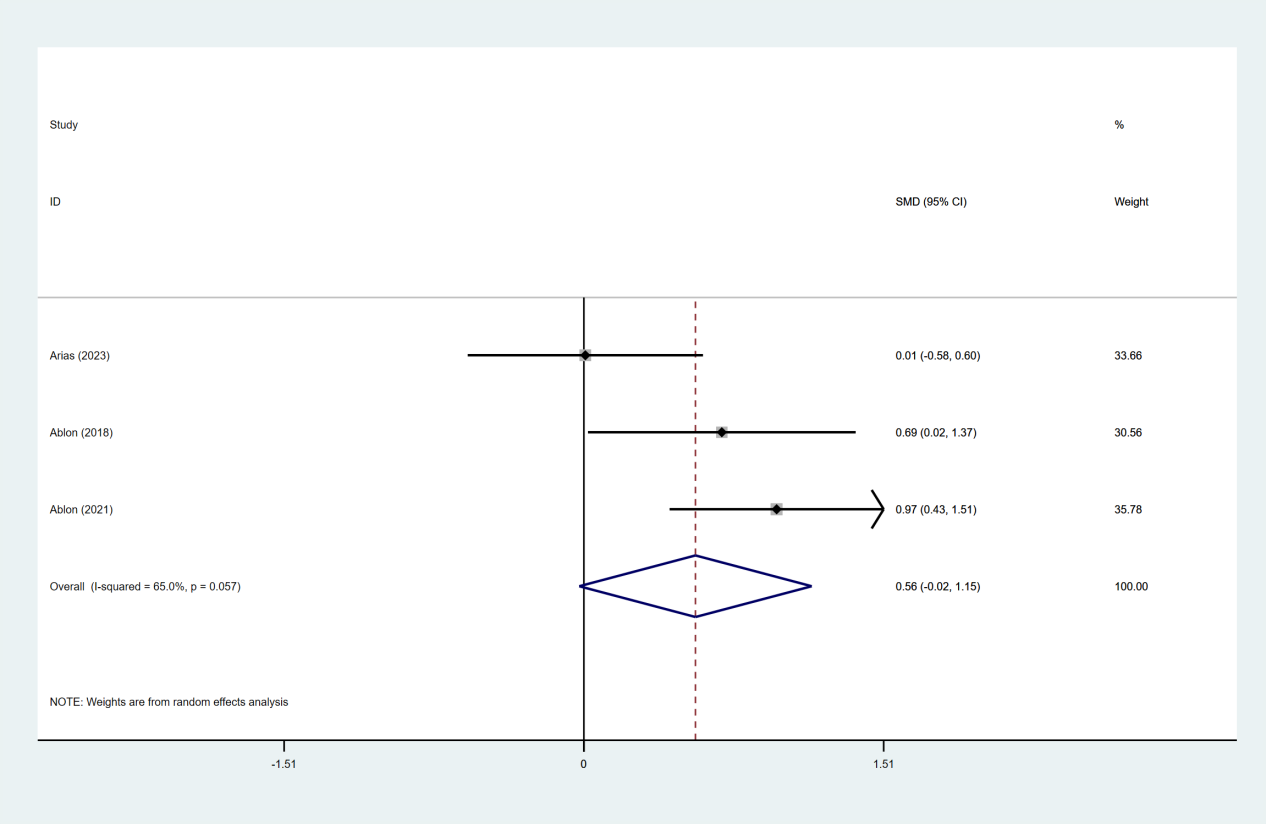


Figure S22 Meta analysis of male subgroup


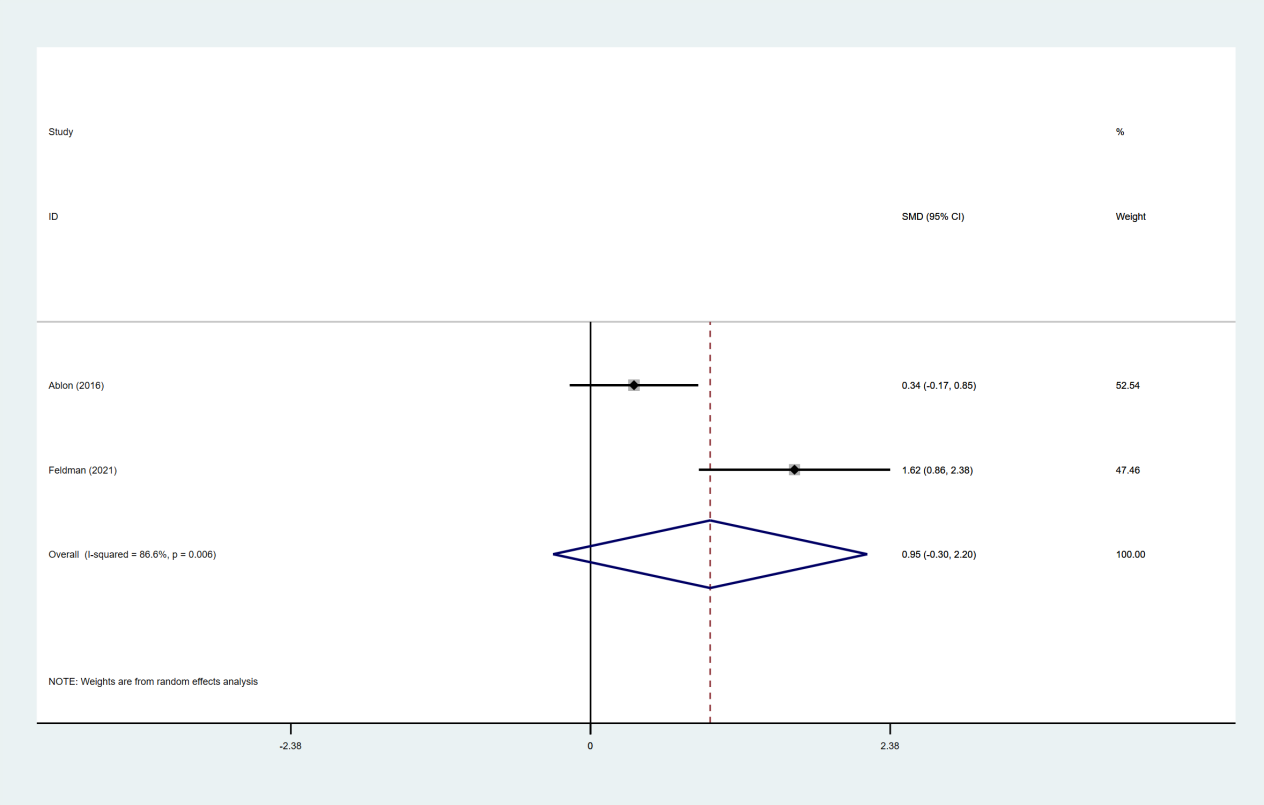


Figure S23 Hair Density subgroup analysis of Non-high-risk


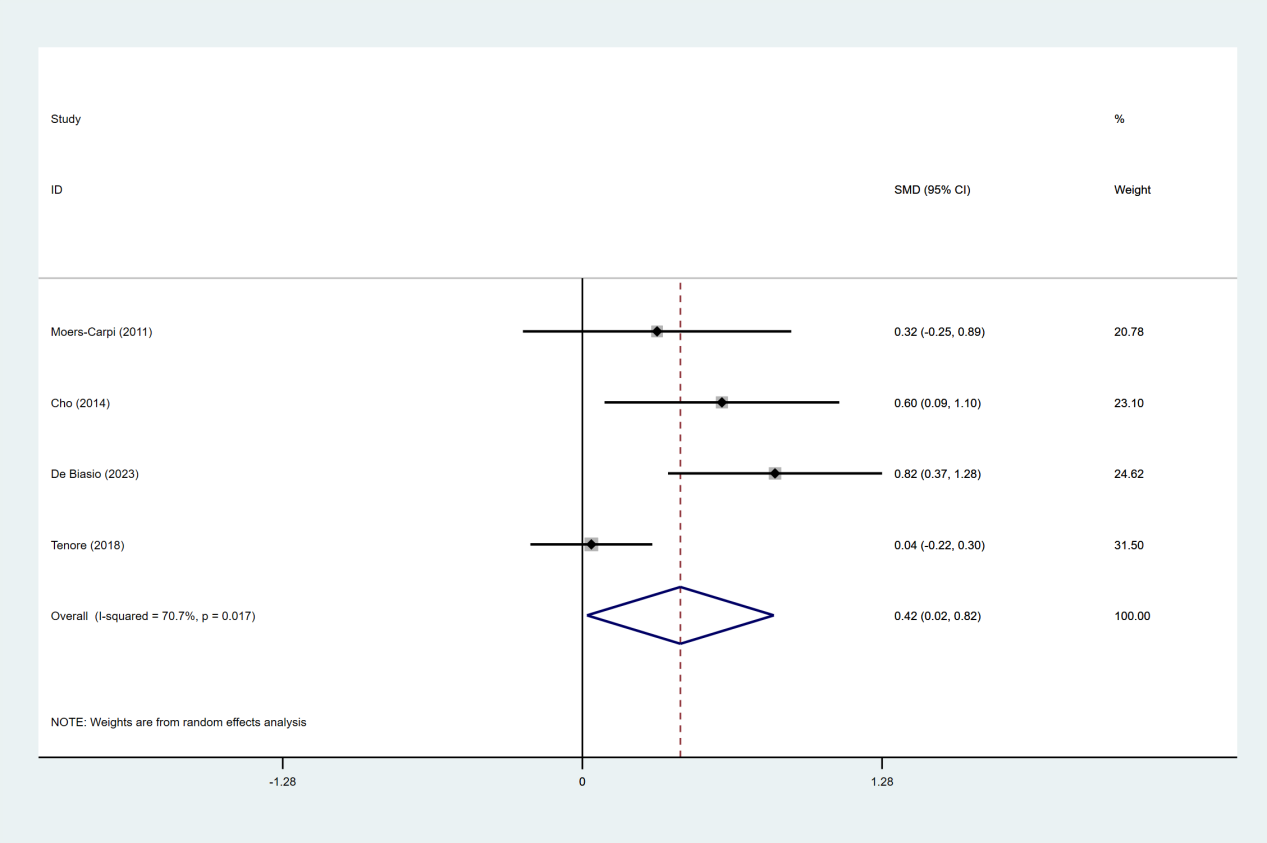


Figure S24 Terminal Hair Density subgroup analysis of Non-high-risk


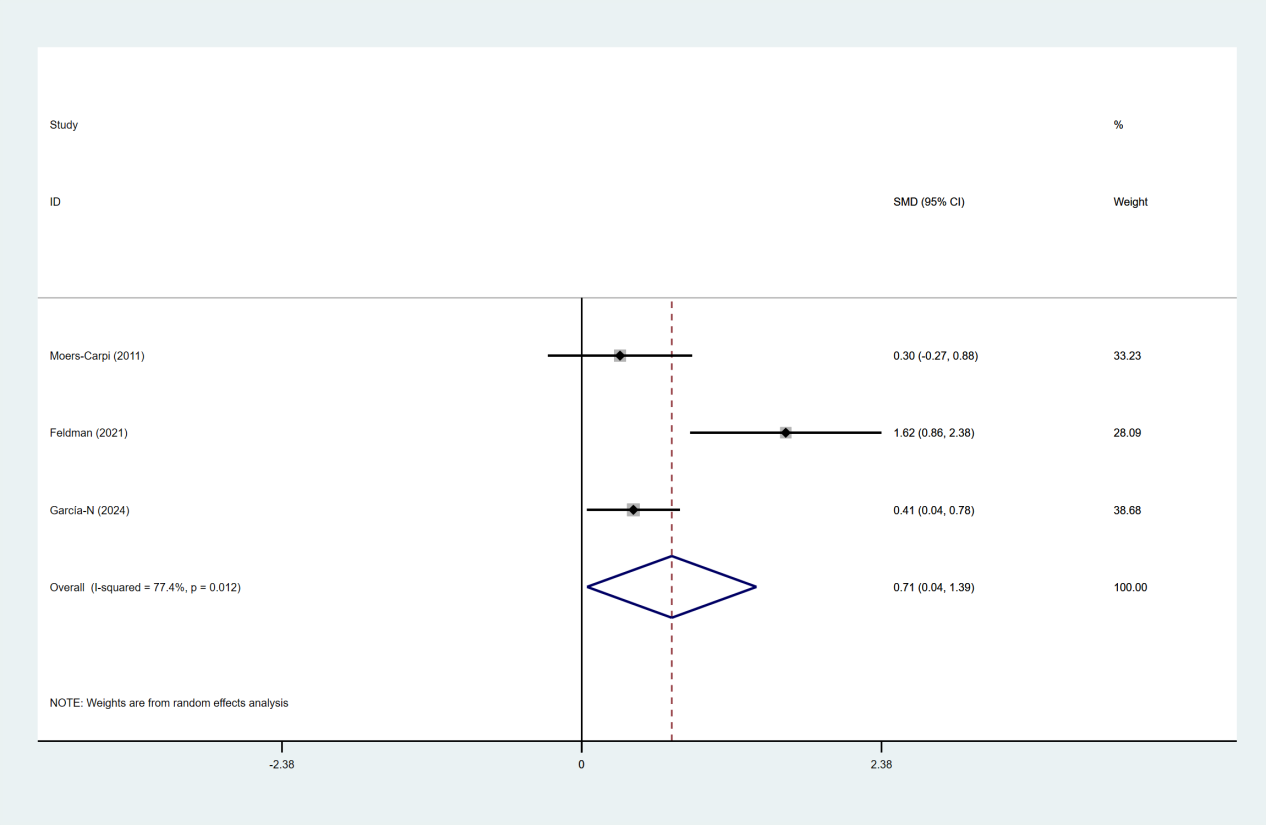


Figure S25 Blind assessment subgroup by doctors analysis of Non-high-risk


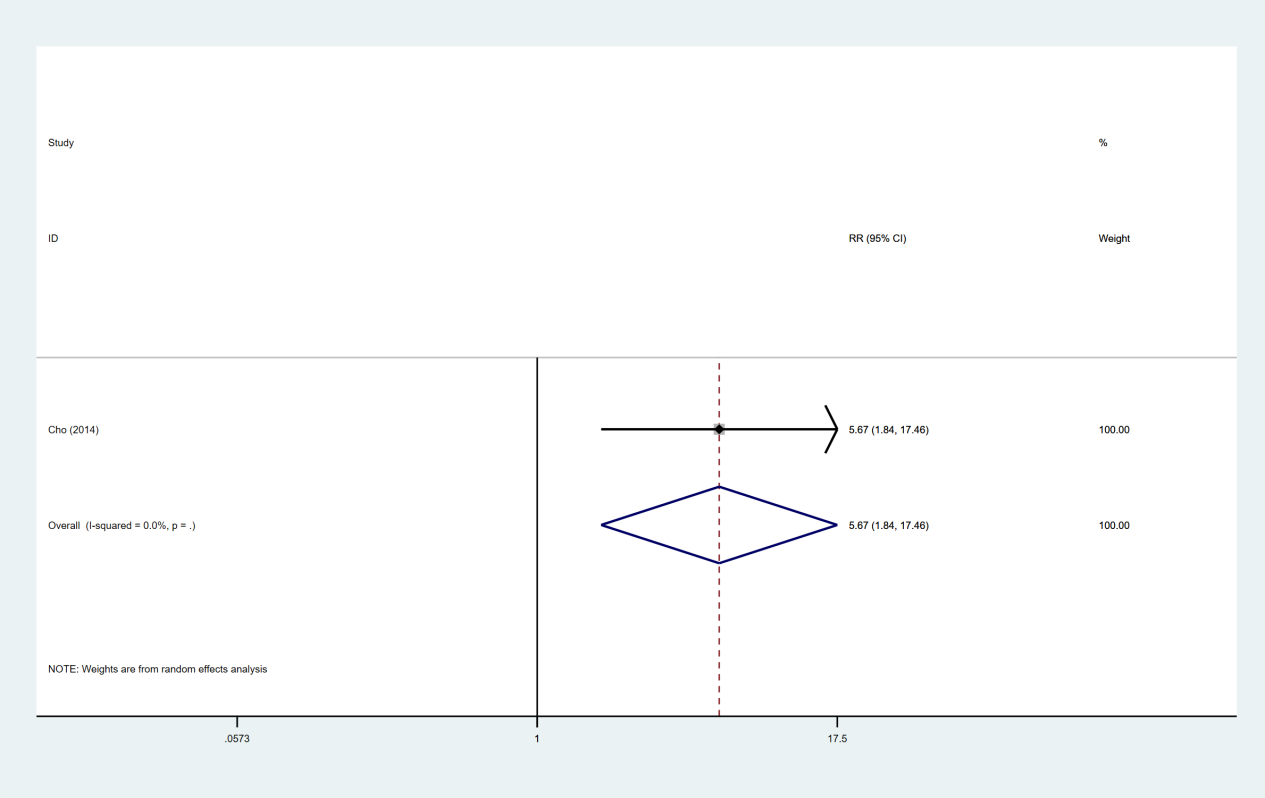


Figure S26 Terminal Hair-to-Vellus Hair Ratio subgroup analysis of Non-high-risk


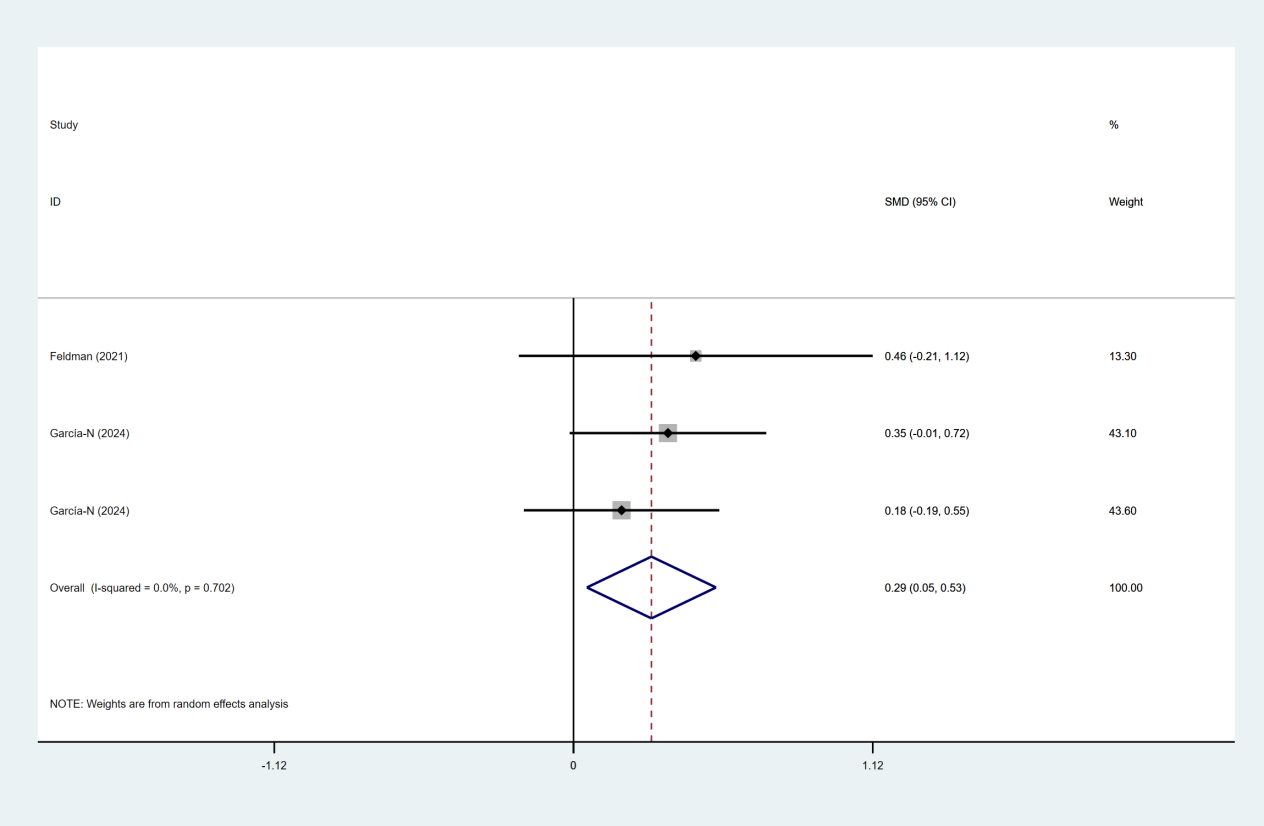


Appendix S1

Search strategy

**Pubmed**

(("Dietary Supplements"[Mesh]) OR ((((((((((((((((((((Dietary Supplements[Title/Abstract]) OR (Dietary Supplement[Title/Abstract])) OR (Supplements, Dietary[Title/Abstract])) OR (Dietary Supplementations[Title/Abstract])) OR (Supplementations, Dietary[Title/Abstract])) OR (Food Supplementations[Title/Abstract])) OR (Food Supplements[Title/Abstract])) OR (Food Supplement[Title/Abstract])) OR (Supplement, Food[Title/Abstract])) OR (Supplements, Food[Title/Abstract])) OR (Nutraceuticals[Title/Abstract])) OR (Nutraceutical[Title/Abstract])) OR (Nutriceuticals[Title/Abstract])) OR (Nutriceutical[Title/Abstract])) OR (Neutraceuticals[Title/Abstract])) OR (Neutraceutical[Title/Abstract])) OR (Herbal Supplements[Title/Abstract])) OR (Herbal Supplement[Title/Abstract])) OR (Supplement, Herbal[Title/Abstract])) OR (Supplements, Herbal[Title/Abstract]))) AND (("Alopecia"[Mesh]) OR (((((((((((((((((((((((Alopecia[Title/Abstract]) OR (Baldness[Title/Abstract])) OR (Hair Loss[Title/Abstract])) OR (Hair Losses[Title/Abstract])) OR (Losses, Hair[Title/Abstract])) OR (Loss, Hair[Title/Abstract])) OR (Pseudopelade[Title/Abstract])) OR (Alopecia Cicatrisata[Title/Abstract])) OR (Alopecia Cicatrisatas[Title/Abstract])) OR (Androgenetic Alopecia[Title/Abstract])) OR (Alopecia, Androgenetic[Title/Abstract])) OR (Pattern Baldness[Title/Abstract])) OR (Baldness, Pattern[Title/Abstract])) OR (Androgenic Alopecia[Title/Abstract])) OR (Alopecia, Androgenic[Title/Abstract])) OR (Alopecias, Androgenic[Title/Abstract])) OR (Androgenic Alopecias[Title/Abstract])) OR (Female Pattern Baldness[Title/Abstract])) OR (Baldness, Female Pattern[Title/Abstract])) OR (Alopecia, Male Pattern[Title/Abstract])) OR (Male Pattern Alopecia[Title/Abstract])) OR (Male Pattern Baldness[Title/Abstract])) OR (Baldness, Male Pattern[Title/Abstract])))

**Embase**

(Alopecia.ti,ab,mh OR "Baldness, Female Pattern".ti,ab OR "Female Pattern Baldness".ti,ab OR "Alopecias, Androgenic".ti,ab OR "Baldness".ti,ab OR "Hairlessness".ti,ab OR "alopecic patch".ti,ab OR "alopecic lesion".ti,ab OR "alopecic disorder".ti,ab OR "alopecic area".ti,ab OR Alopecia.mh) AND ( "dietary supplements".mh OR "nutritional supplements".ti,ab OR "dietary supplements".ti,ab OR "food supplements".ti,ab OR "herbal supplements".ti,ab OR vitamin.ti,ab OR "folic acid".ti,ab OR mineral.ti,ab OR probiotic.ti,ab OR "fish oil".ti,ab OR "co - enzyme q10".ti,ab OR "amino acid".ti,ab OR "cherry extract".ti,ab OR "celery seed extract".ti,ab OR turmeric.ti,ab)

**Cochrane Library**

( ( "Dietary Supplements":ti,ab,kw OR "Nutriceuticals":ti,ab,kw OR Nutraceutical:ti,ab,kw OR "Neutraceuticals":ti,aid,kw OR "Nutraceuticals":ti,ab,kw OR "Nutriceutical":ti,ab,kw OR "Neutraceutical":ti,ab,kw OR "Supplement, Herbal":ti,ab,kw OR "Supplements, Herbal":ti,ab,kw OR "Herbal Supplements":ti,ab,kw OR "Herbal Supplement":ti,ab,kw OR "Dietary Supplementations":ti,ab,kw OR "Supplementations, Dietary":ti,ab,kw OR "Supplement, Food":ti,ab,kw OR "Dietary Supplement":ti,ab,kw OR "Food Supplement":ti,ab,kw OR "Food Supplementations":ti,ab,kw OR "Supplements, Dietary":ti,ab,kw OR "Food Supplements":ti,ab,kw OR "Supplements, Food":ti,ab,kw OR dietary supplement:mesh ) AND ( "Alopecia":ti,ab,kw OR "Alopecia Cicatrisata":ti,ab,kw OR "Alopecia Cicatrisatas":1,ab,kw OR "Pseudopelade":ti,ab,kw OR "Alopecia, Androgenic":1,ab,kw OR "Androgenic Alopecias":ti,ab,1,kw OR Alopecia, Androgenetic:1,ab,kw OR Baldness, Pattern:1,ab,kw OR "Androgenetic Alopecia":ti,ab,kw OR "Alopecias, Androgenic":1,ab,kw OR "Androgenic Alopecia":1,ab,kw OR "Pattern Baldness":1,1,kw OR "Loss, Hair":1,ab,kw OR Losses, Hair:1,ab,kw OR Hair Losses:1,ab,kw OR Hair Loss:1,ab,kw OR "Baldness":ti,ab,kw OR "Alopecia, Male Pattern":1,ab,kw OR "Male Pattern Alopecia":1,ab,kw OR "Male Pattern Baldness":1,1,kw OR "Baldness, Male Pattern":1,ab,kw OR Female Pattern Baldness:1,ab,kw OR Baldness, Female Pattern:1,ab,kw ) )

**Web of Science**

((TS=(Dietary Supplements) OR TS=(Supplements, Dietary) OR TS=(Dietary Supplementations) OR TS=(Supplementations, Dietary) OR TS=(Food Supplementations) OR TS=(Food Supplements) OR TS=(Food Supplement) OR TS=(Supplement, Food) OR TS=(Supplements, Food) OR TS=(Nutraceuticals) OR TS=(Nutraceutical) OR TS=(Nutriceuticals) OR TS=(Nutriceutical) OR TS=(Neutraceuticals) OR TS=(Neutraceutical) OR TS=(Herbal Supplements) OR TS=(Herbal Supplement) OR TS=(Supplement, Herbal) OR TS=(Supplements, Herbal))AND (TS=(androgenetic alopecia) OR TS=(Baldness) OR TS=(Hair Loss) OR TS=(Hair Losses) OR TS=(Losses, Hair) OR TS=(Loss, Hair) OR TS=(Pseudopelade) OR TS=(Alopecia Cicatrisata) OR TS=(Alopecia Cicatrisatas) OR TS=(Androgenetic Alopecia) OR TS=(Alopecia, Androgenetic) OR TS=(Pattern Baldness) OR TS=(Baldness, Pattern) OR TS=(Androgenic Alopecia) OR TS=(Alopecia, Androgenic) OR TS=(Alopecias, Androgenic) OR TS=(Androgenic Alopecias) OR TS=(Female Pattern Baldness) OR TS=(Baldness, Female Pattern) OR TS=(Alopecia, Male Pattern) OR TS=(Male Pattern Alopecia) OR TS=(Male Pattern Baldness) OR TS=(Baldness, Male Pattern) OR TS=(Alopecia)))
